# Technological innovations on direct carbon mitigation by ordered energy conversion and full resource utilization

**DOI:** 10.1007/s43979-022-00009-5

**Published:** 2022-04-19

**Authors:** Liejin Guo, Zhisong Ou, Ya Liu, Zhiwei Ge, Hui Jin, Guobiao Ou, Mengmeng Song, Zihao Jiao, Wenhao Jing

**Affiliations:** State Key Laboratory of Multiphase Flow in Power Engineering, Xi’an Jiaotong University, No. 28, Xianning West Road, Xi’an, 710049 People’s Republic of China

**Keywords:** Carbon neutrality, Coal utilization, Direct carbon mitigation, Supercritical water gasification, Renewable energy, CO_2_ reduction

## Abstract

Coal consumption leads to over 15 billion tons of global CO_2_ emissions annually, which will continue at a considerable intensity in the foreseeable future. To remove the huge amount of CO_2_, a practically feasible way of direct carbon mitigation, instead of capturing that from dilute tail gases, should be developed; as intended, we developed two innovative supporting technologies, of which the status, strengths, applications, and perspective are discussed in this paper. One is supercritical water gasification-based coal/biomass utilization technology, which orderly converts chemical energy of coal and low-grade heat into hydrogen energy, and can achieve poly-generation of steam, heat, hydrogen, power, pure CO_2_, and minerals. The other one is the renewables-powered CO_2_ reduction techniques, which uses CO_2_ as the resource for carbon-based fuel production. When combining the above two technical loops, one can achieve a full resource utilization and zero CO_2_ emission, making it a practically feasible way for China and global countries to achieve carbon neutrality while creating substantial domestic benefits of economic growth, competitiveness, well-beings, and new industries.

## Introduction

The extensive consumption of fossil fuels, deforestation and other forms of anthropogenic activities are leading to an increase of greenhouse gas concentration in the atmosphere, causing approximately 1.0 °C of global warming above pre-industrial levels [1, 2]. The consequently severe weather and natural disasters, as well as environmental pollutions, are threatening human’s survival. Meanwhile, the increasing energy demand and limited fossil fuel supply are exacerbating energy security issues as well as geopolitical instabilities. To tackle the climate crisis and construct a low-carbon sustainable society, participating countries in the Paris Climate Summit held in 2015 (COP21 climate change summit) declared an agreement on holding the global average temperature increase well below 2 °C, preferably to 1.5 °C within this century. More than 120 countries have successively proposed their carbon neutral plans: the major industrialized countries, most European Union countries, the US, etc., aim to achieve the target mostly by 2050 [3, 4]; China is the leading developing country striving to the carbon mitigation, pledging to reach carbon peak before 2030 and achieve carbon neutralization by 2060 [5]. The decarbonization plan would bring profound benefits to the environmental sustainability [6, 7], air quality [8–11], and human’s health; however, the related social impacts and technical constraints pose challenges to the rapid transition towards a carbon-neutral society from a fossil-based industrial and economic system [7, 12, 13].

At present, six greenhouse gases need to be carefully regulated, including carbon dioxide (CO_2_), methane, nitrous oxide, hydrofluorocarbons, perfluorocarbons, and sulfur hexafluoride. Significantly, CO_2_ is the largest portion of greenhouse emissions. The PBL greenhouse emission report shows that in 2020 the proportion of CO_2_ and methane in global greenhouse gas emissions is 73% and 19%, respectively [14]. The proportion of nitrous oxide is 5%, and the others accounts for 3%. According to British Petroleum (BP) statistics (PETROLEUM–BP 2020), global carbon emission has increased by 40% from 2000 to 2019. In 2019, the global carbon emissions reached 34.36 billion tons, which is the highest point in history. Due to the impact of COVID-19, in 2020, global carbon emissions dropped by 6.3% to 32.28 billion tons. Specifically, in 2020, China’s carbon emissions reached 9.899 billion tons, which consists mainly of (I) production and supply of electric power, steam, and hot water, (II) smelting and pressing of ferrous metals, (III) nonmetal mineral products, (IV) transportation, storage, post and telecommunication services, and others. Among them, the energy consumption within industrial production accounts for the main part of carbon emission.

According to the International Energy Agency (IEA), the CO_2_ emissions from coal account for 44% and 79.5% of total emission, globally and in China, respectively. Some countries have been intended to phase down coal utilization as their concrete plans, as widely discussed in [15–19]; however, it is not a practically feasible option in China from near-term perspective: among the total coal consumption in China, nearly 51% of coal is used in coal-fired plants, generating over 70% of annual electricity, which is impossible to be suddenly substituted with the intermittent and random renewable power from energy security consideration [12, 20, 21]; the other 49% of coal is consumed mostly for heating or used as resources in industrial sectors, which could only be changed if there are economically feasible non-fossil substances that can be widely used as alternatives (but it seems impossible at present) [20, 22]. Even that carbon capture and storage (CCS) techniques seem to be a promising way to remove those fossil carbon mitigation, they are seldom deployed in an expected scale due to the lower-than-desired efficiency [23], as well as causing obvious energy efficiency loss and extra expenses. The dilemma of the huge amount of CO_2_ emission from fossil consumption and increasing demands of energy in the economic development requires a widespread and urgent transformation and innovations in the energy-related industry.

From the perspective of energy and resource utilization, current fossil consumption systems behave in a disordered manner: the mismatch between energy conversion and material transformation leads to high energy dissipation and material dispersion. This can be exemplified through the coal-fired energy utilization system: the chemical energy of coal is converted into thermal energy through coal combustion with air, then the heated water steam goes through turbines and pushes power generator for electricity production; the multiple heat transfer processes cause considerable heat dissipation and efficiency decreases, and the thermal-mechanical conversion governed by Carnot Cycle further leads to huge energy wastes [24]; besides, the collocated chemical elements in coal and air are converted into dispersed products, such as CO_2_, nitrogen oxide (NO_x_), sulfur oxide (SO_x_), particulate matters (PM), harmful organics, heavy metals, and minerals [25–28], either the gathering or purification requires huge energy consumption, and inevitably leads to the energy loss and a waste of resources, particularly the CO_2_ and pollutant emissions.

The present paper discussed the philosophy and progress of direct carbon mitigation approaches, which is the research focus of the authors’ group in State Key Laboratory of Multiphase Flow in Power Engineering (SKLMFPE). Within such approaches, the fossil resources can be efficiently and orderly conversed, while the CO_2_ can be naturally gathered as resources for renewable fuel production, leading to a carbon-neutral fossil utilization system. This article hereafter is organized as follows: Section 2 gives the landscape of the fossil-based carbon-neutral system; Section 3 and 4 discuss two concrete technological innovations developed in SKLMFPE, the supercritical water gasification (SCWG) technology for coal utilization and renewables-power fuel production techniques via CO_2_ reduction reaction (CO_2_RR), respectively; Section 5 presents the perspective of the above approaches; at last, some conclusions are drawn in Section 6.

## Coal-based carbon-neutral system by ordered energy conversion and full resource utilization

**Fig. 1:**
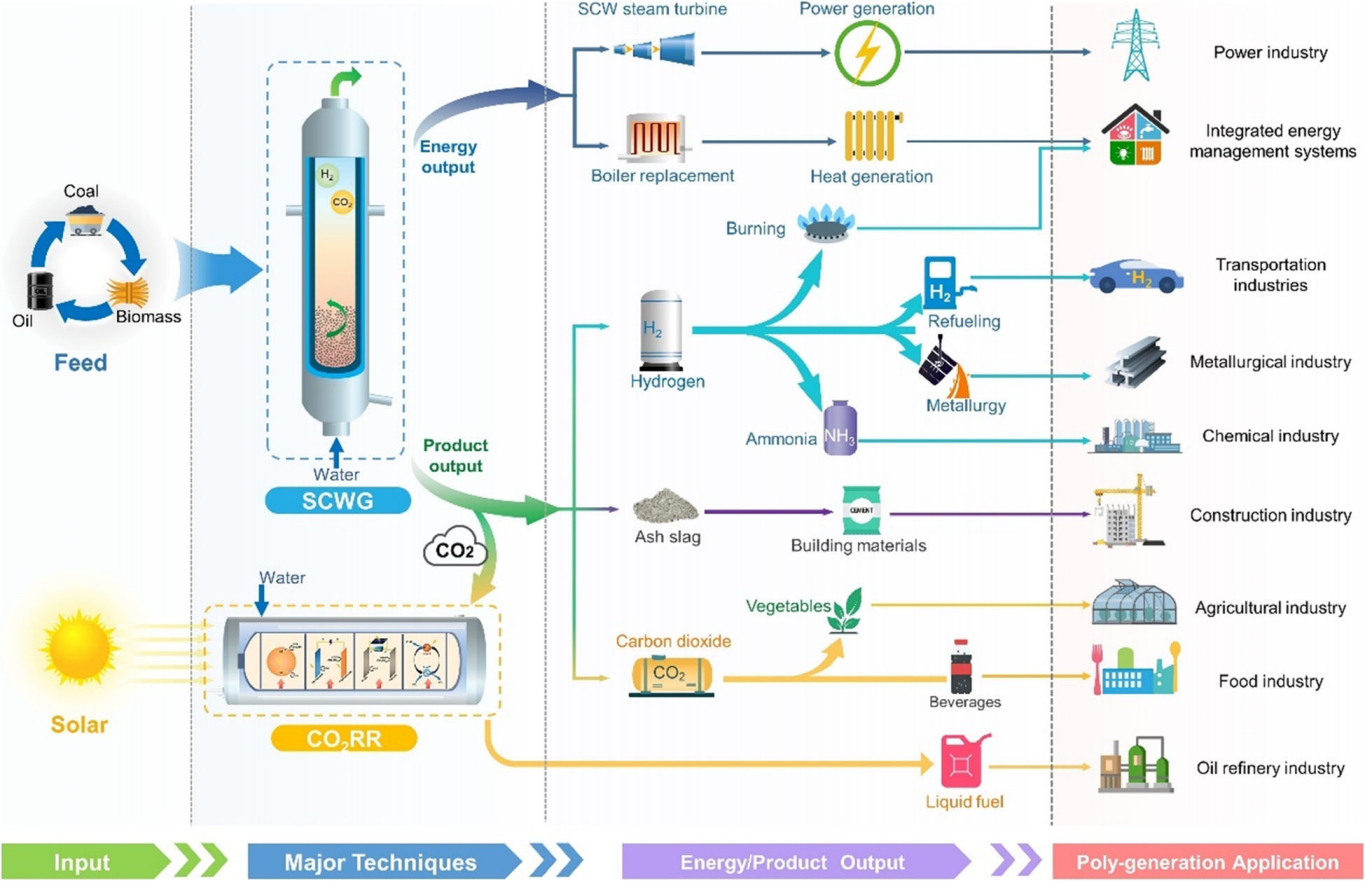
Schematic of carbon-neutral system based on SCWG-based poly-generation and renewables-powered CO_2_RR technique

To eliminate the huge CO_2_ emission from fossil-based energy production, an innovative coal (or other carbon-containing sources, such as biomass and oil) utilization technology based on supercritical water gasification is developed in SKLMFPE, as shown in Fig. 1. The carbon-based resource is gasified in a reductive supercritical water (SCW) atmosphere into H_2_ and CO_2_ as well as minerals (slag).

According to the end-use demands, the poly-generation can be achieved: (1) for hydrogen production, gas products can be further separated for purified hydrogen in a low energy consumption way; (2) for heat (or power) generation, part of (or all) hydrogen can be oxidized in a mild environment, generating steam for heating (or pushing steam turbines); (3) combining the above two methods for hydrogen-heat-electricity production. Once combined with the CO_2_RR process, the CO_2_ produced in the above SCWG-based poly-generation processes, which is naturally in a gathered and high-purity state, can be easily utilized for carbon-based fuel production, leading to direct carbon mitigation of fossil utilization. Apart from gas products, the nitrogen, sulfur, metal elements and various minerals in coal can be gradually purified and deposited in the form of slag with the conversion of coal. The solid slags can be used as building materials, while the metal elements can be further purified and used as metallic resources. Based on the above techniques, an ideal fossil utilization system is constructed: all the resources are fully used without waste generation and CO_2_ emission.

In view of energy conversion, the overall SCWG-based thermal processes keep in a mild temperature (generally lower than 700 °C), having a much less exergy loss than the traditional coal combustion process. Besides, the reheat of steam is finished in an in-situ state with hydrogen oxidation, avoiding the energy loss due to indirect heat transfer between different parts of the heat exchanger. The energy utilization system thus has an overwhelming advantage in extremely high energy efficiency, as will be discussed later. From long-term consideration, the sustainable and green energy supply is a necessary solution, but the randomness and intermittence of carbon-free energy (e.g., solar, wind and geothermal) restricted the further deployment; such bottleneck is effectively released through renewables-powered CO_2_RR process, which converts the unstable renewable energy into chemical energy of carbon-based fuels. The overall energy conversion system behaves in an ordered way, and works towards maximum efficiency and the practically feasible transition to sustainable energy.

## Poly-generation based on SCWG of coal: innovational coal utilization, and large-scale and low-cost hydrogen and electricity production

Coal will still play a dominant role in ensuring the stability of electrical grid as well as serving as resources in end-use sectors in a long period [22, 29]. The combustion-based utilization mode leads to high energy consumption, heavy pollutions (e.g., SO_x_, NO_x_, and PM), and enormous CO_2_ emissions [27, 30, 31]; and it brings challenges to human’s health, environmental sustainability, and global ecosystem [32]. Reducing carbon emission in coal conversion is the major carbon mitigation task towards carbon neutralization. This section introduces a novel coal utilization technology based on supercritical water gasification (SCWG), which converts the chemical energy of coal and the low-grade heat to high-purity hydrogen [33–35] and produces high-purity CO_2_ for carbon-based fuel production. This process eliminates pollutant generation in combustion processes [33–36], and achieves zero CO_2_ emission through combining with renewable-powered CO_2_ techniques. The mechanism, status, challenges, and technological breakthroughs of SCWG are introduced in this section; as intended, we discuss the possibilities in achieving poly-generations as well as strengths in realizing direct carbon mitigation compared to coal combustion.

### Principles of supercritical water gasification

In 1978, Modell first reported the phenomenon that high heating value gaseous products can be obtained from organic solids and liquids via supercritical water treatments [37], known as supercritical water gasification (SCWG). Since then, SCWG has been extensively studied around the world, as discussed in [38–46]. Different from the traditional combustion/gasification process, SCWG uses supercritical water (SCW, with temperature greater than 374.15 °C and pressure greater than 22.1 MPa) as the reaction medium, and the chemical energy of organic matters can be effectively converted into H_2_-rich gas mixtures in a state that can be easily captured [47]. Ge et al. [48] studied the coal gasification characteristic, and achieved the complete gasification of organic compounds (oxy-hydrocarbons) in coal in a micro batch reactor at 700 °C, while the inorganic components (pure ash in the original state) are left as slag. This process can be represented by

$$ \mathrm{Coal}+{H}_2O\overset{P\ge 22.1 MPa,\kern0.5em T\ge 374{}^{\circ}C}{\to }{H}_2+{CO}_2+{Ash}_{pure}. $$

Compared with coal combustion, SCWG of coal has several major differences: 1) the SCWG process undergoes in a relatively low operating parameter (less than 700 °C), which ensures a safer plant operation and enables a possibly lower capital cost; 2) coal is treated in a reductive SCW atmosphere, eliminating the generation of SO_x_, NO_x_, and other harmful substances; 3) the CO_2_ is generated in a high pressure state, which can be naturally collected without extra carbon capture processes, and is valuable resource instead of greenhouse gas emission. Apart from the above advantages, SCWG is attractive in fast chemical reaction performance and high energy transformation efficiency, which are decisional towards industrial application and are discussed in this subsection.

#### Transport properties and reactant behavior of SCW

**Fig. 2:**
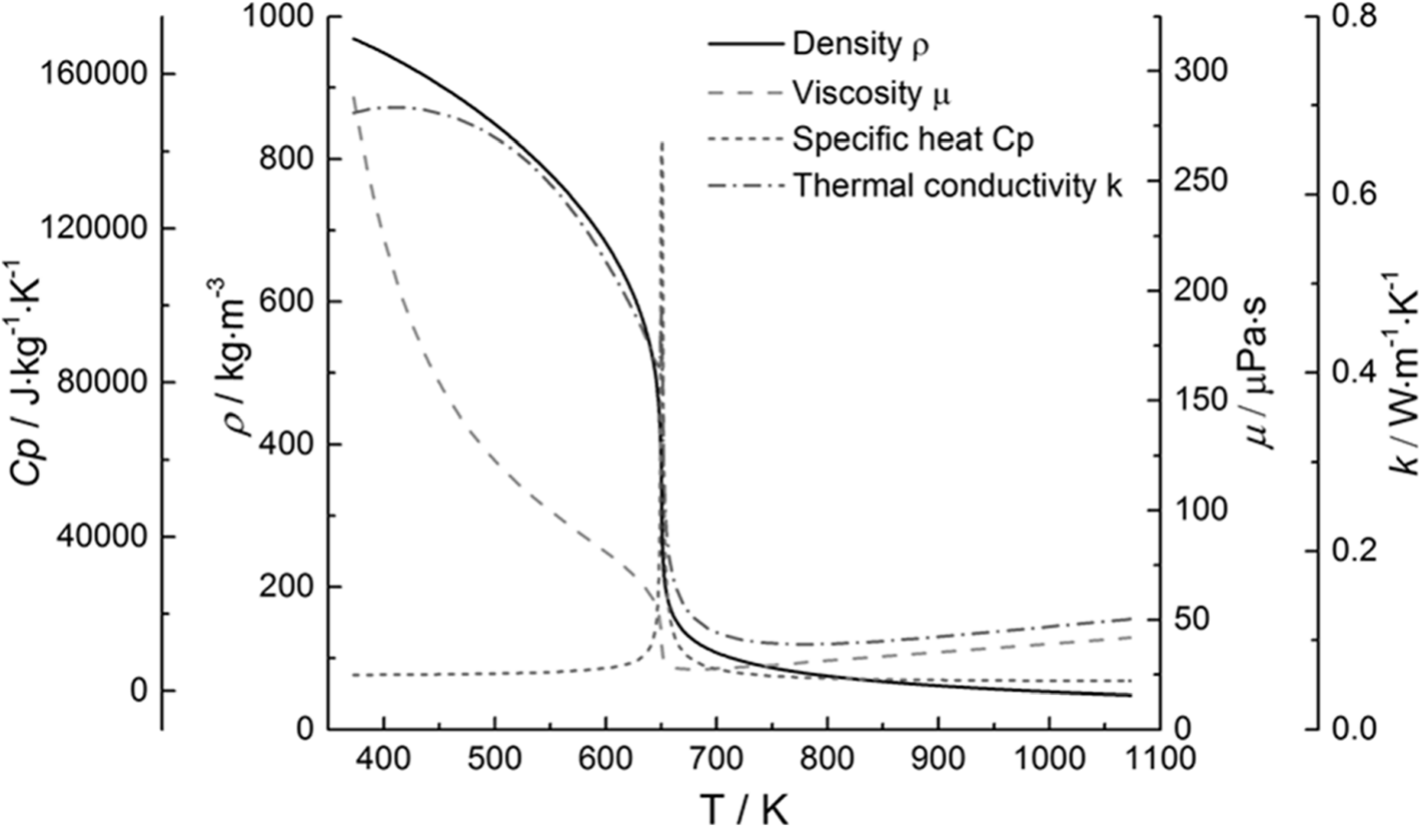
Thermophysical and transport properties of supercritical water, based on NIST Database [49]

The transport properties of water change drastically as the temperature increases (as shown in Fig. 2), behaving both like liquids and steams: the density of water decreases dramatically across the critical point, but it is still two orders of magnitude higher than that of steam, showing advantages of high reactant concentration; besides, the gas-like low viscosity significantly improves the diffusivity of water, and reduces the mass transfer resistances during the reaction processes.

Owing to the above features, SCW is regarded as an ideal reaction medium for conversion of coal, biomass, and organic wastes [50–53]. When water is heated to supercritical state, the breakdown of hydrogen bonds among water molecules due to the decreased density leads to the dramatic decrease of dielectric constant [47], making SCW an reliable solvent for nonpolar organics. Additionally, the decrease of the ion product further triggers the free-radical reactions, promoting the efficient conversion of coal particles into gaseous products [54].

#### Reaction mechanism and optimization of SCWG

**Fig. 3:**
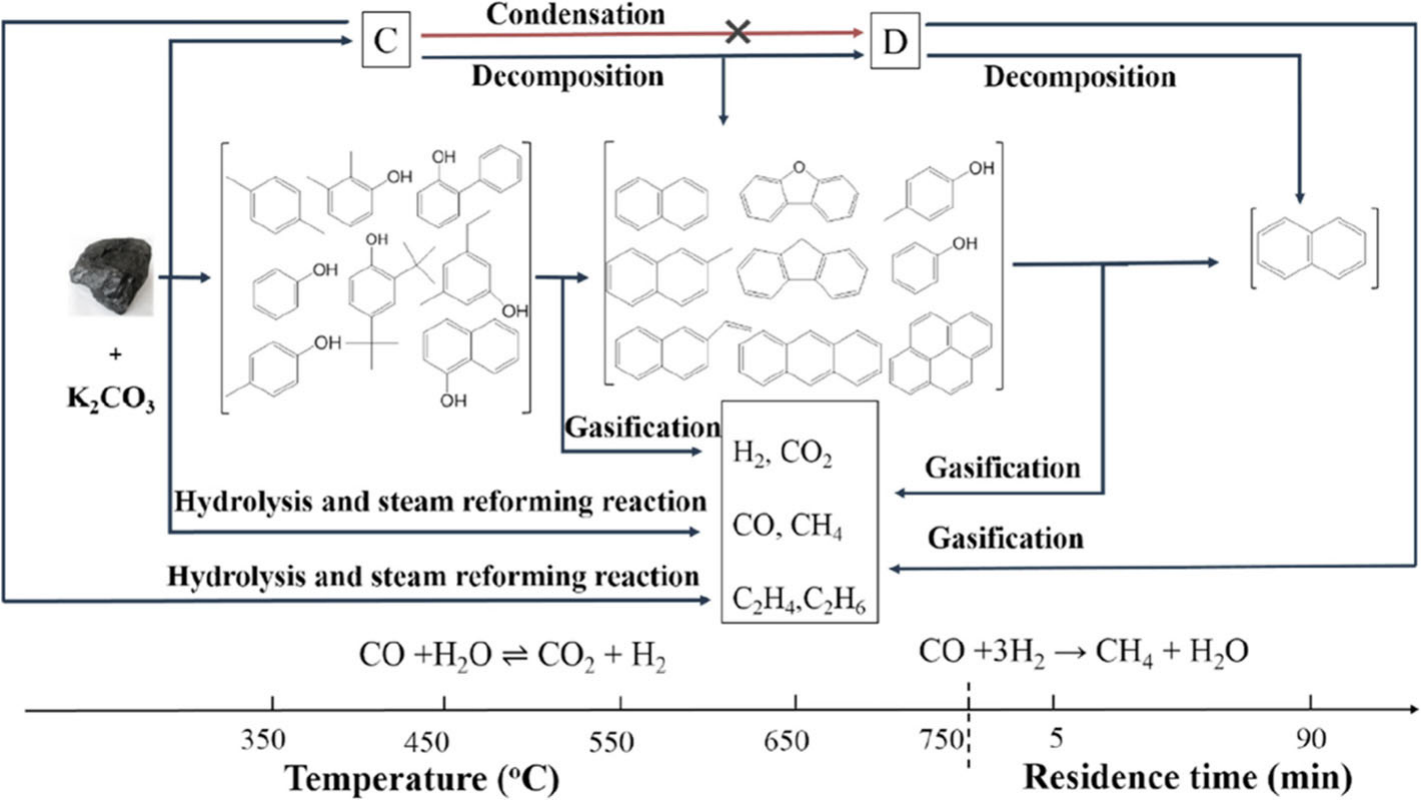
Conversion mechanism of SCWG of Zhundong coal with K_2_CO_3_; 3 wt%, 17.19–25.95 MPa (residence time starts when heated to 750 °C). C denoted solid products from the massive breakage of cross-linked bonds between aromatic structures, and from violent hydrolysis of coal with K_2_CO_3_. D denoted the solid products from the decomposition of large aromatic structures in product C. Reprinted from [56]

During the coal gasification process, many intermediates are generated: part of intermediates are directly decomposed into small gaseous fragments through hydrolysis reaction; while, other large fragments of aromatics which contains strong bonding energy are converted into H_2_ and CO_2_ through heterogeneous reactions [55]. Sun et al. [56] systematically studied the pathway of SCWG (as shown in Fig. 3), and found that the graphite-like structure, i.e., polycyclic aromatic hydrocarbons (PAH) generated during the heating process, is stable in SCW; thus, the gasification of PAH is the rate-controlling step to achieve the complete coal gasification in SCW. To overcome the reaction barrier, Liu et al. [57], Zhu et al. [55], and Sun et al. [56] further studied various homogeneous catalysts, and found that K_2_CO_3_ is effective in promoting the coal hydrolysis and aromatics decomposition, and in suppressing the graphite-like PAHs’ production [56].

To reveal the mechanism of catalytic reaction, Wang et al. [58] studied the mechanism of carbon-water reaction using theoretical approaches, and concluded that: the alkali can strengthen the strong-chemical adsorption of water on carbon structure, thus can inhibit the polymerization between aromatics. Furthermore, several experimental studies have confirmed that the addition of alkali, especially K_2_CO_3_, can effectively enhance the gasification efficiency of coal, as discussed in Ref. [55, 57, 59–63].

### Key engineering techniques for SCWG

Apart from the chemical reaction kinetics, the interphase heat and mass transfers are other factors affecting the reaction equilibrium towards efficient hydrogen production [64, 65]. Many efforts have been made to get enhanced interphase interaction in the past decades, and various reactors has been developed. Generally, the reactors can be classified into batch reactors [66–68] and continuous reactors [69–71]. The improper matching of chemical reaction and interphase heat or mass transfer in batch reactors exacerbates the side reactions and leads to an incomplete gasification, as presented in Ref. [37, 72–79].

**Fig. 4:**
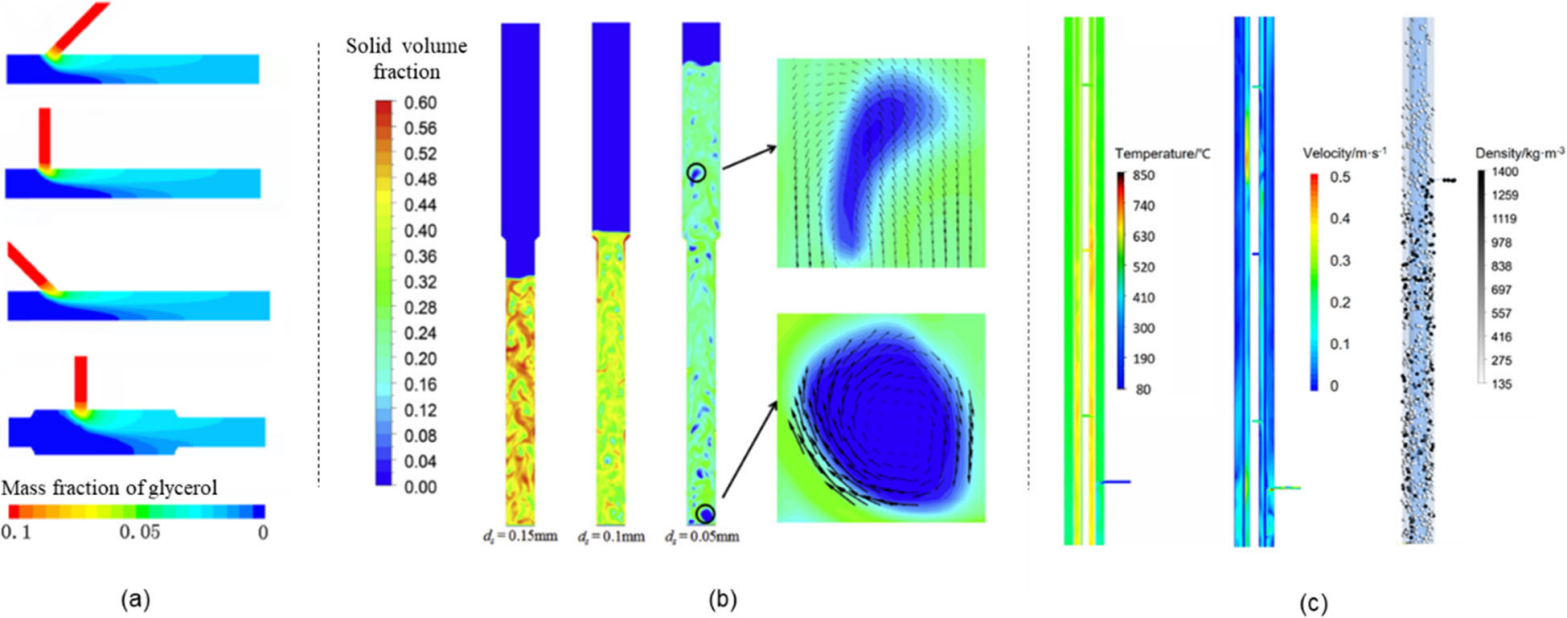
SCWG reactor: **a** tabular reactor; **b** fluidized-bed reactor; **c** thermally coupled reactor. Reprinted from [92–94], respectively

For better reaction performance, the authors’ group developed various continuous reactors, as shown in Fig. 4. In tubular reactors, the homogeneous feedstocks (e.g., glucose, glycerol, and organic wastewater) can effectively gasified; however, the mismatch of flow direction and gravity leads to insufficient dispersing, and causes clogging in solids processing, as shown in Fig. 4(a). Lu et al. [80–82] studied the flow dynamics of mono-diameter Geldart-B particles in supercritical water flows both experimentally and numerically [83–86], and proposed the fluidization theories [82, 87–89]; subsequently, the supercritical water fluidized bed reactor is developed [90, 91] typically as shown in Fig. 4(b), within which the feedstocks have a rapid contacting with fresh SCW thus can be effectively transformed into gas products. The newly developed thermally coupled reactor (in Fig. 4c) further achieves the efficient energy supply to the endothermic coal gasification regions from exothermic hydrogen oxidation chambers [92].

Other breakthroughs have been made in developing continuous coal slurry transporting and slug discharging system, both of which are multiphase transporting process and are limited by the high operation parameters (25 MPa, 650 °C). Ren et al. and Ou et al. [95, 96] studied the trans-critical injection characteristics of coal slurries into SCWG reactors, and obtained the optimized conditions for continuous coal slurry supply and dispersing. Based on that, we developed a multi-channel nozzle with cooling device, and achieved the continuously feeding of the coal slurry. Cheng et al. [97] studied the pressure-driven particle-laden flows, and developed a step-down pressure-driven slug discharging system which can effectively remove the solid residues out of reactor.

**Fig. 5:**
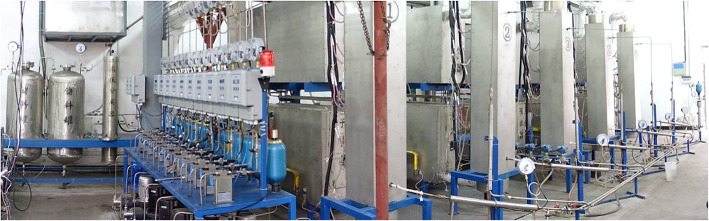
Experimental demonstration plant in SKLMFPE with 5-paralleled module

Based on the above-mentioned technological breakthroughs, an experimental demonstration plant with 5-paralleled module is established to realize the efficient gasification of coal under mild temperature [98], as shown in Fig. 5. The typical gasification results from 72-h continuously running are shown in Table 1, indicating that coal can be effectively gasified into H_2_ and CO_2_, with a yield of 1.55 Nm^3^ and 0.93 Nm^3^ per kilogram of standard coal, respectively. In all the experimental studies, a variety of coals are tested, almost all of which can be well treated in a wide range of coal parameters (such as moistures, ash content, the ash melting point, etc.), showing extremely well adaptabilities. Furthermore, Jin et al. [63] reported that an equivalent gasification efficiency performance can be achieved in a much lower operating parameter (at 530 °C, 25 MPa) with external recycle system, making the SCWG more promising towards industrial-scale application.

**Table 1:** Gas composition and yields of small-scale SCWG system, reproduced from [98]

Gas composition	H_2_	CO	CH_4_	CO_2_	C_2_H_6_
Volume fraction (%)	55.20	0.64	10.26	33.17	0.71
Gas yield (Nm^3^/kg coal)	1.55	0.02	0.29	0.93	0.02

**Fig. 6:**
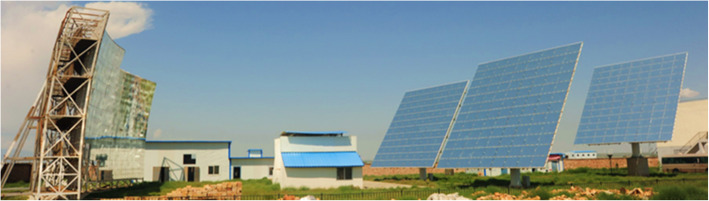
Pilot-scale demonstration plant of SCWG system driven by concentrated solar energy, reprinted from [34]

For treating other carbon-containing resources, our group has achieved many progresses, such as in efficient biomass gasification [70, 99], in effective removal of organics in wastes (e.g., sewage sludge [100, 101] and black liquor [102, 103]), and in degradation of plastics [104, 105]. These processes can be further empowered with renewables, for which we established a pilot-scale demonstration project for SCWG of biomass coupled with solar heating in Ningxia Hui Autonomous Region, China (in Fig. 6) [106]. The designed feeding rate of the system is 1.03 tons per hour, and the reacting unit can operate at 800 °C and 40 MPa. For the heating system, a maximum power of 163 kW can be achieved by concentrated solar heating, and the thermal efficiency of the reactor reaches 73.1%.

In continuous operation of the system, a typical testing condition is carried out: the 5 wt.% corn cob slurry is treated at a flow rate of 270 kg/h in the system, and the solar radiation intensity is 776 W/m^2^. The main gaseous products consist of H_2_ and CO_2_, and the percentages can reach 44.4% and 39.5%, respectively. All the energy are from renewables in such system, and the feedstock can be effectively gasified, proving the feasibility of the system scaling-up and that SCWG can be possibly deployed for treating a wide range of resources, in combination with renewables-powered heat supply [34].

### SCWG-based poly-generation in industrial applications

**Fig. 7:**
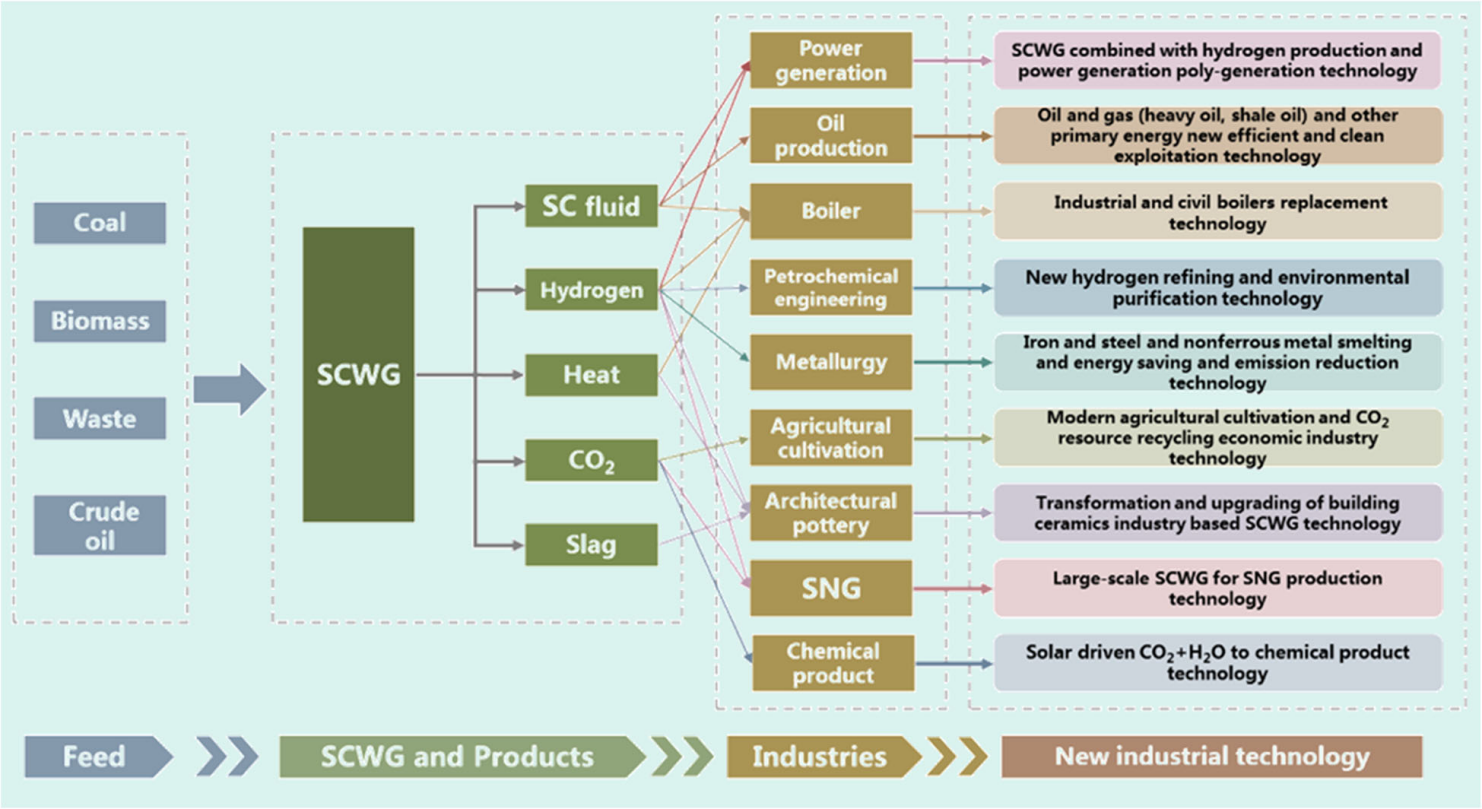
Upgradation and transformation of traditional industries by SCWG-based poly-generation

The SCWG process provides a wide variety of products including steam, heat, H_2_, CO_2_, and mineral slags; when combined with end-use sectors, many possibilities can be achieved in upgrading traditional energy industries, as well as developing new industries (as indicated in Fig. 7). According to the industrial demands, the hydrogen can either be used through mild oxidation for heat and power generation, or directly used as fuel and chemical resources; other products are naturally in a high-pressure gathered state, thus can be easily used as valuable resources instead of wasting.

Towards industrial-scale applications of SCWG, many on-going demonstrating projects are being conducted in SKLMFPE. Herein, we introduce three representative industrial demonstration projects including the scenery for poly-generation of hydrogen and heat, for power generation, and for power generation and ammonia production.

#### Poly-generation for hydrogen and heat generation

**Fig. 8:**
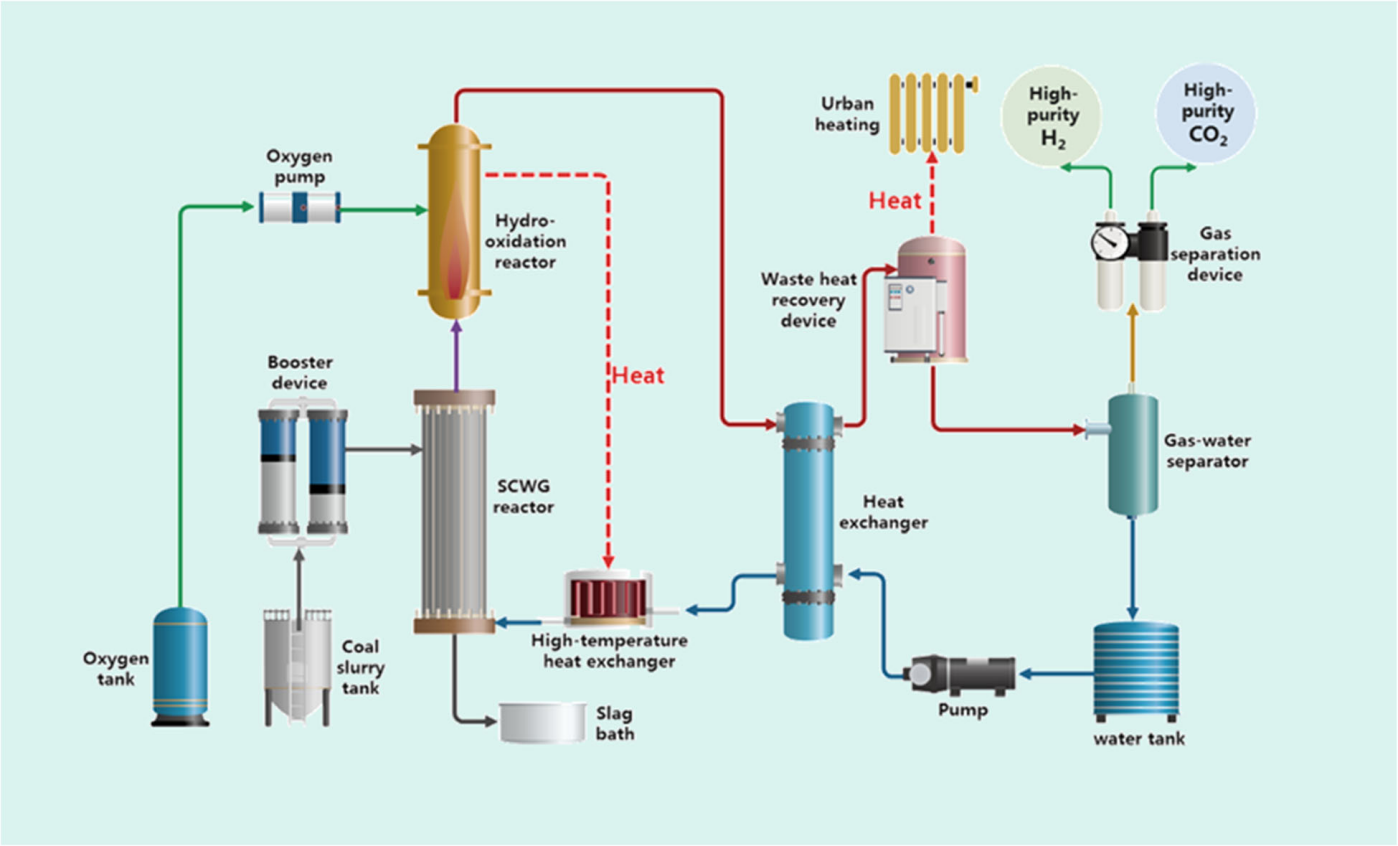
System schematic of poly-generation for hydrogen and heat

Given the target of hydrogen and heat supply (widely used in hydrofining and other industries), a hydrogen production system is designed, as shown in Fig. 8. The coal can be efficiently converted to hydrogen-rich gas in the SCW reactor, and then hydrogen-rich products react with oxygen moderately in the supercritical hydro-oxidation reactor to generate mixed working medium and heat. Subsequently, the mixed working medium exchanges heat with the high-pressure water, and the residual heat is recovered by the waste heat recovery device for the heating or the generation of low-pressure steam. Finally, the water is separated through a pressure-reduction device for recycling, while the gas products enter the gas separation unit for high-purity hydrogen and CO_2_ production.

**Fig. 9:**
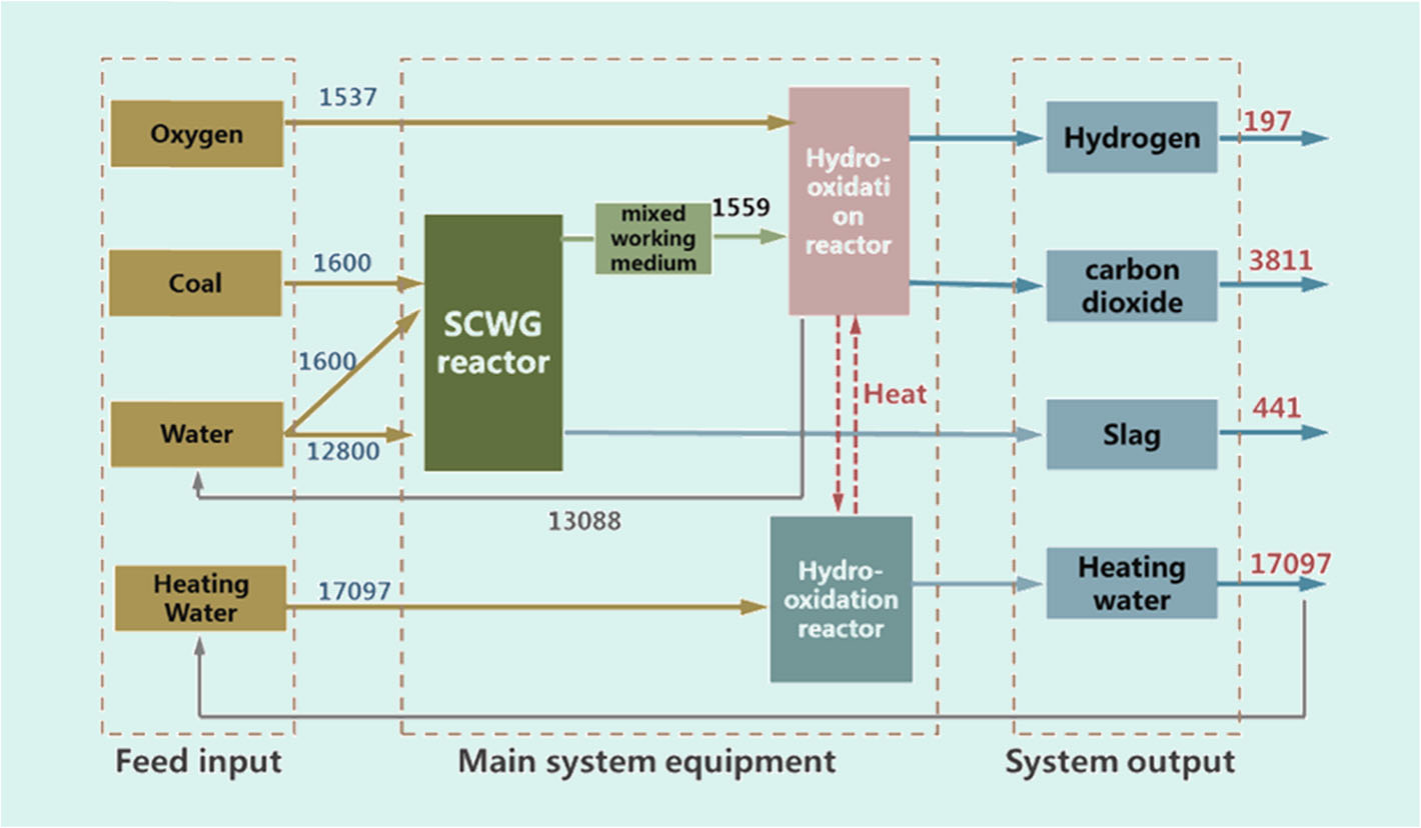
Material balance of 2000 Nm^3^-scale SCWG-based hydrogen production system (kg/h)

The material balance of the system is shown in Fig. 9. Taking a 2000 Nm^3^-scale hydrogen production system for example, it consumes 1.6 tons of dry coal (3.2 tons of 50 wt.% coal slurry) and 1.54 tons of oxygen per hour, and produces 197 kg of high-purity hydrogen, 3.8 tons of high-purity CO_2_, and 0.44 tons of ash per hour. According to the energy calculation, the calorific value of coal consumed is 10.456 MW, the calorific value of hydrogen produced is 5.955 MW, and the heat recovered by waste heat is 3.218 MW. The efficiency of hydrogen production, hydrogen-heat cogeneration, and hydrogen-heat-gas cogeneration are 54.31%, 83.66%, and 93.58%, respectively.

**Fig. 10:**
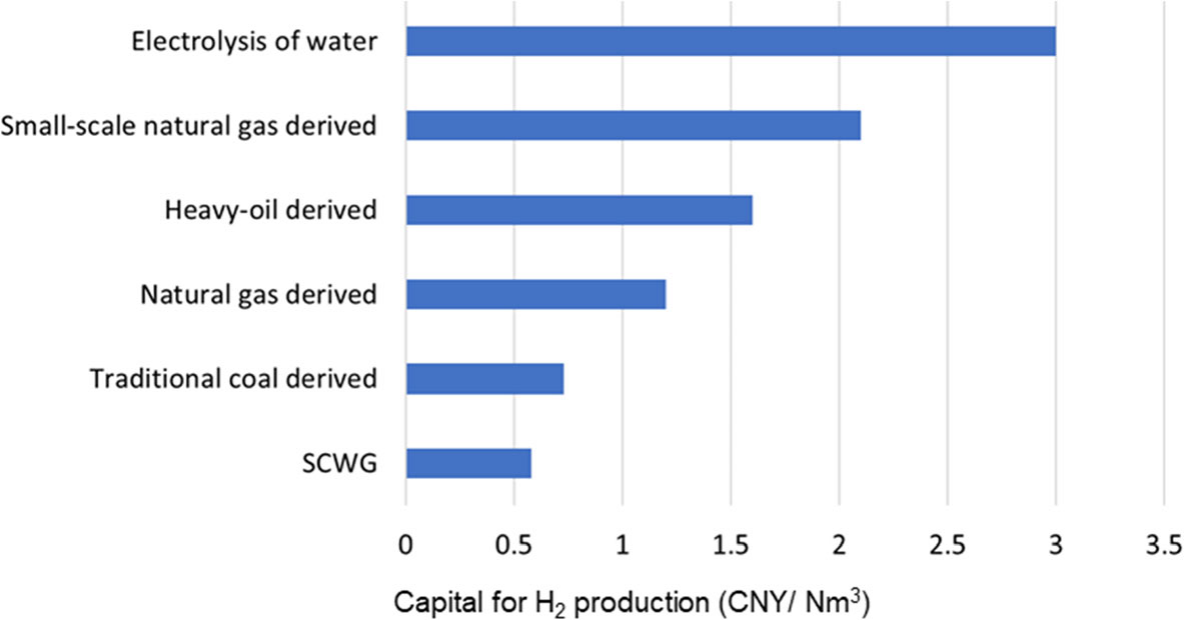
Economic analysis of SCWG-based H_2_ production, in comparison with other techniques

When compared with other hydrogen production technologies, the SCWG-based hydrogen production cost can be lowered down to 0.58 CNY/Nm^3^, which is much lower than traditional approaches (as shown in Fig. 10), providing a practically feasible way for large-scale and low-cost hydrogen production. Furthermore, the high-pressurized H_2_ produced in SCWG can be directly used as chemical resources, e.g., for ammonia [107] and methanol [108, 109] production, without high energy consumption in the gas compression process, benefiting in energy and cost saving.

#### SCWG-based power generation

**Fig. 11:**
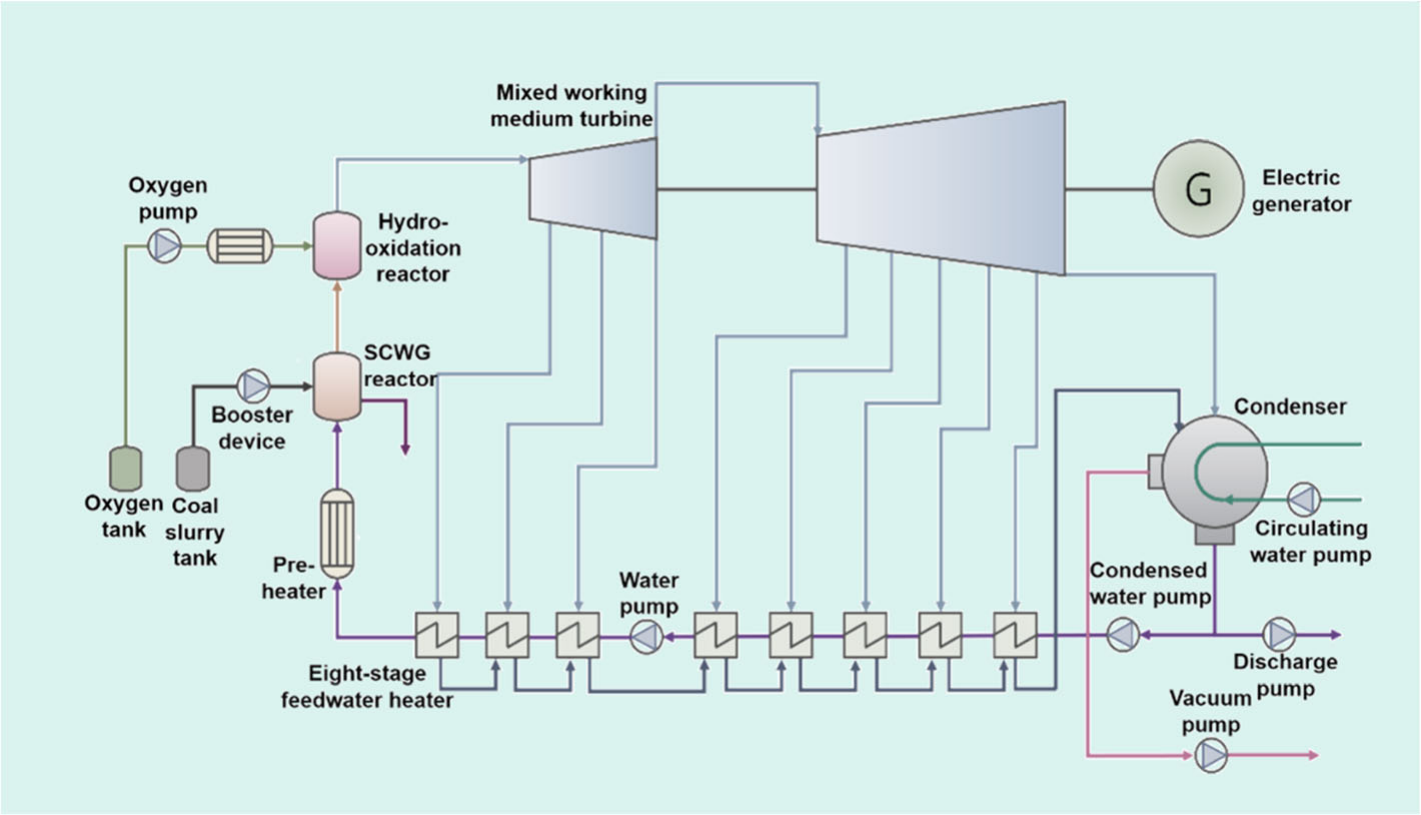
Schematic of SCWG-based poly-generation system for power generation

Similar as the hydrogen production system, a maximum power generation can be achieved by oxidizing all the hydrogen in the supercritical hydro-oxidation reactor. A typical poly-generation system is proposed for such purpose as shown in Fig. 11. The system mainly consists of SCWG submodule, mixed-working-medium turbine and its auxiliary submodule, CO_2_ collection and phase separation submodule, etc. It should be noted that the SCWG thermal power generation system is in open cycle condition, with the oxidation products being a part of the mixed working fluid. Besides, the mixed working medium is non-azeotropic and its temperature drops continuously during condensation process; therefore, the irreversibility of heat transfer process can be reduced by matching temperature slip characteristics of mixed working medium. The system adopts one reheat and eight regenerations, and the cycle efficiency of mixed working medium can reach 50% under typical operating conditions. Finally, CO_2_ can be continuously enriched in the gas phase during the condensation process and can be separated at low cost for further utilization.

**Fig. 12:**
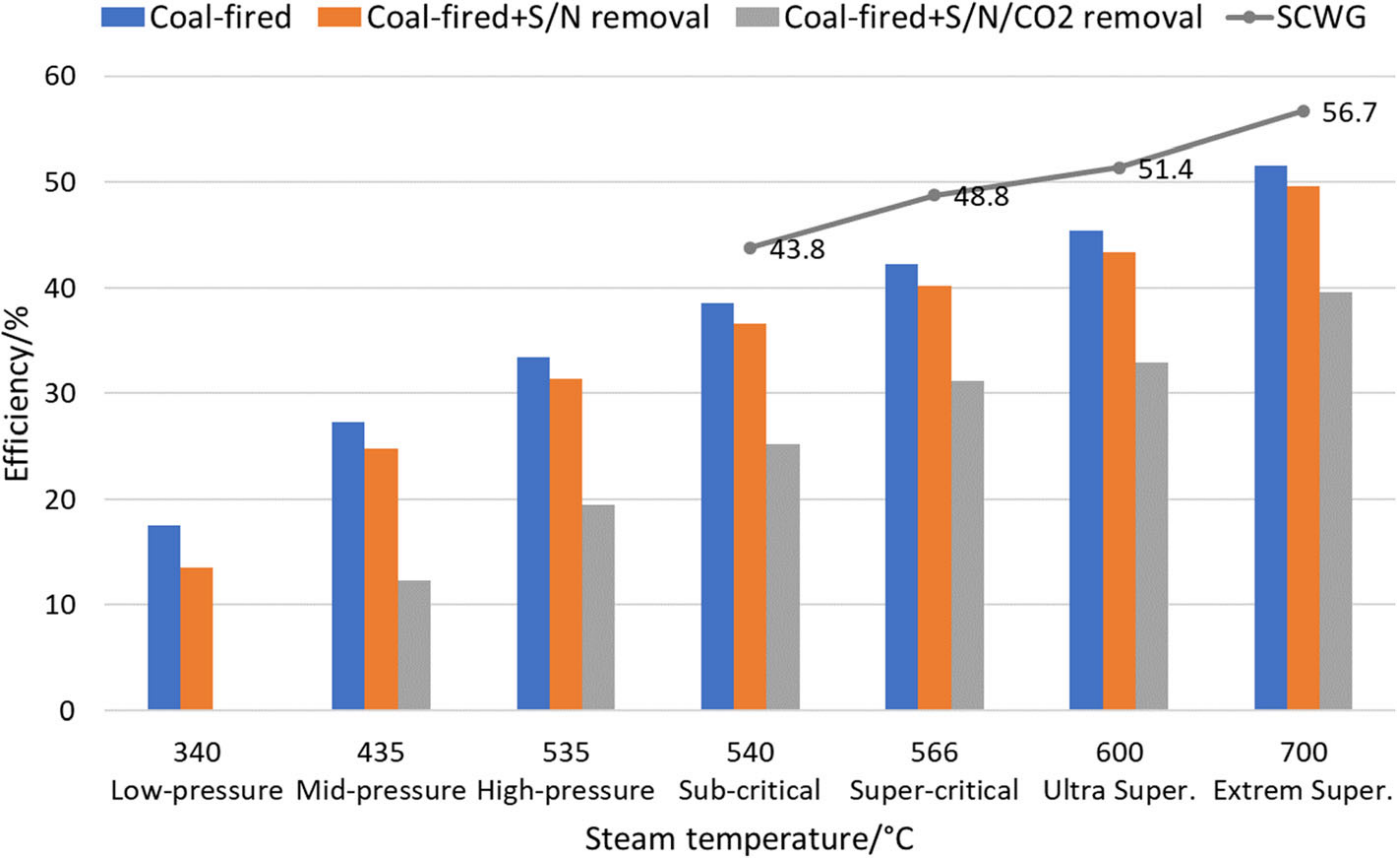
Comparison of SCWG-based power generation efficiency with other strategies

In traditional coal-fired power generation, the power generation efficiency increases with the increase of steam temperature, as shown in Fig. 12. Coal combustion inevitably causes huge emissions of pollutants (e.g., SO_x_, NO_x_, and PM_2.5_) and CO_2_ (more than 750 g CO_2_ emission per kilowatt for a 1000 MW-scale power station, and higher for smaller scales). The cleaning process for SO_x_, NO_x_, and PM_2.5_ removal requires huge energy consumption, leading to 2% ~ 4% energy efficiency loss; the power efficiency further decreases for 10% ~ 13% if CCS-based techniques are used for the CO_2_ removal.

**Fig. 13:**
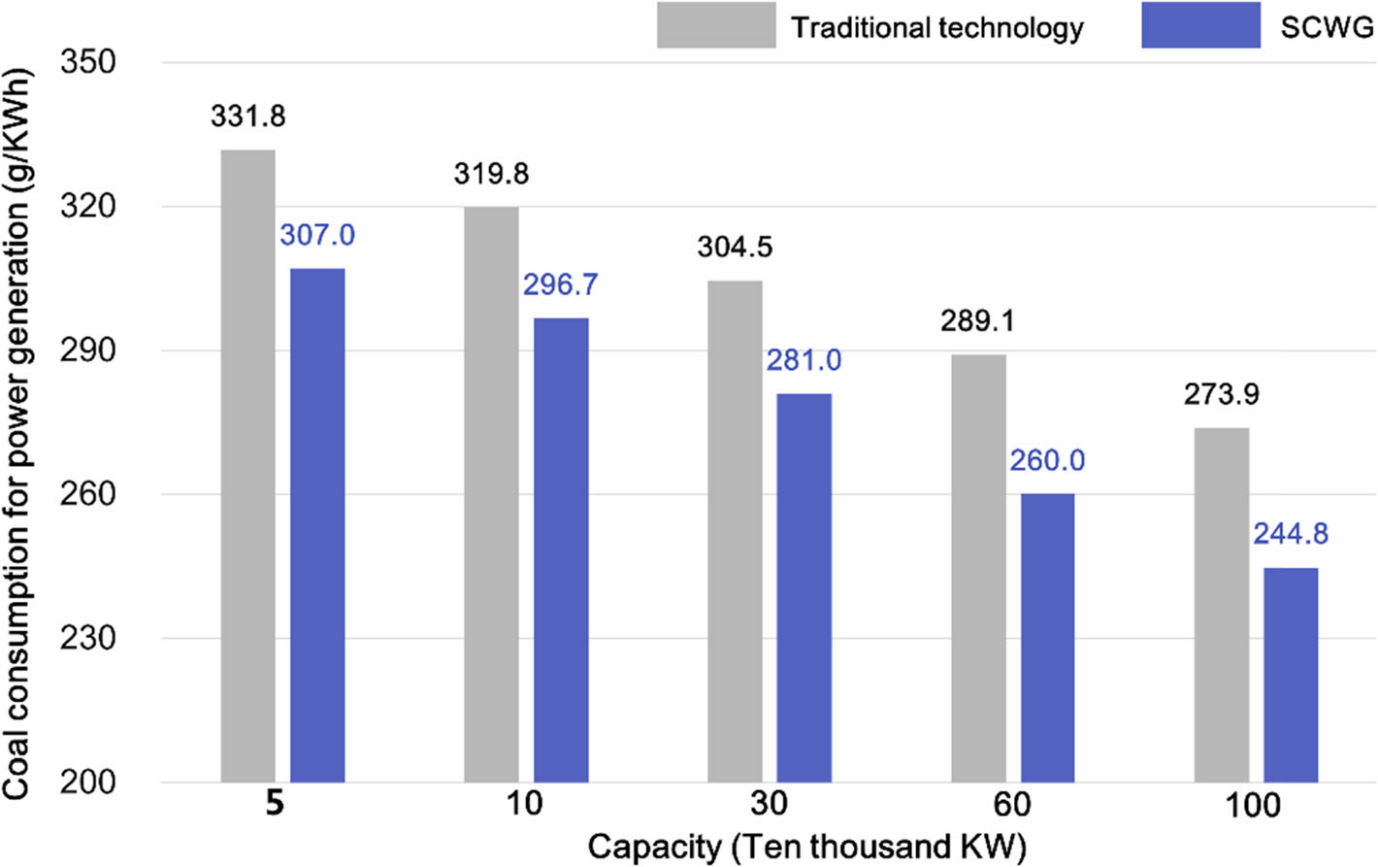
Coal consumption rate for power generation (SCWG vs. traditional way)

A comparison of the coal consumption between traditional coal-fired power generation and SCWG is conducted, as shown in Fig. 13. SCWG have a less coal consumption in a wide range of power capacity; when the power generation capacity reaches 1000 MW, the SCWG-based coal consumption rate decreases to a 244.8 g/kWh, saving coal of 29.1 g/kWh compared with traditional thermal power generation, and the power generation efficiency reaches 56.7% (in Fig. 12).

**Fig. 14:**
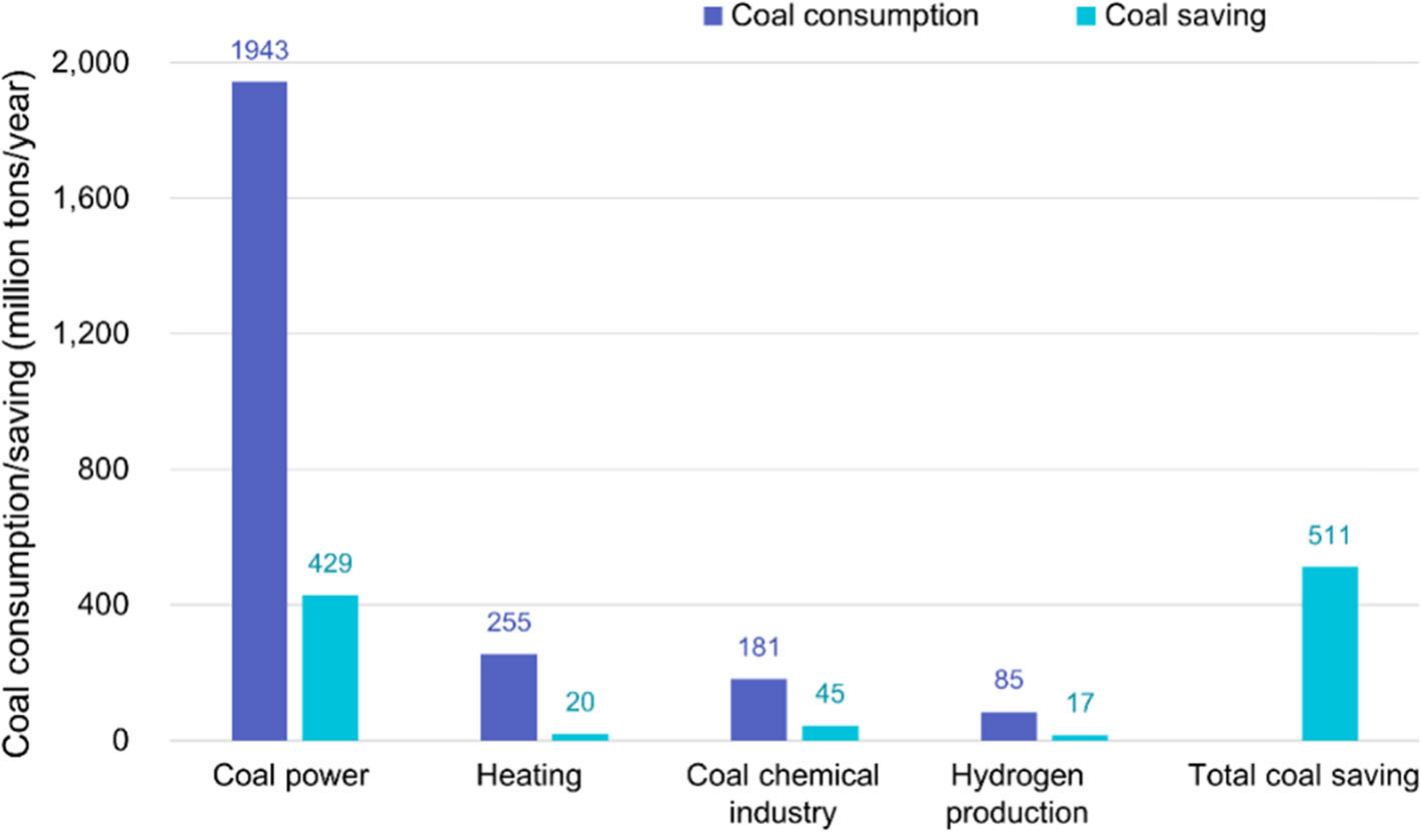
Coal consumption and coal savings in various industries by SCWG-based technology

Further, if SCWG-based technology is fully extended and applied in China, an enormous coal saving can be achieved in power generation, heating, chemical engineering, hydrogen production, and other industrial fields. According to the annual coal consumption in different sectors, a potential coal saving of 511 million tons per year can be achieved (as shown in Fig. 14).

**Fig. 15:**
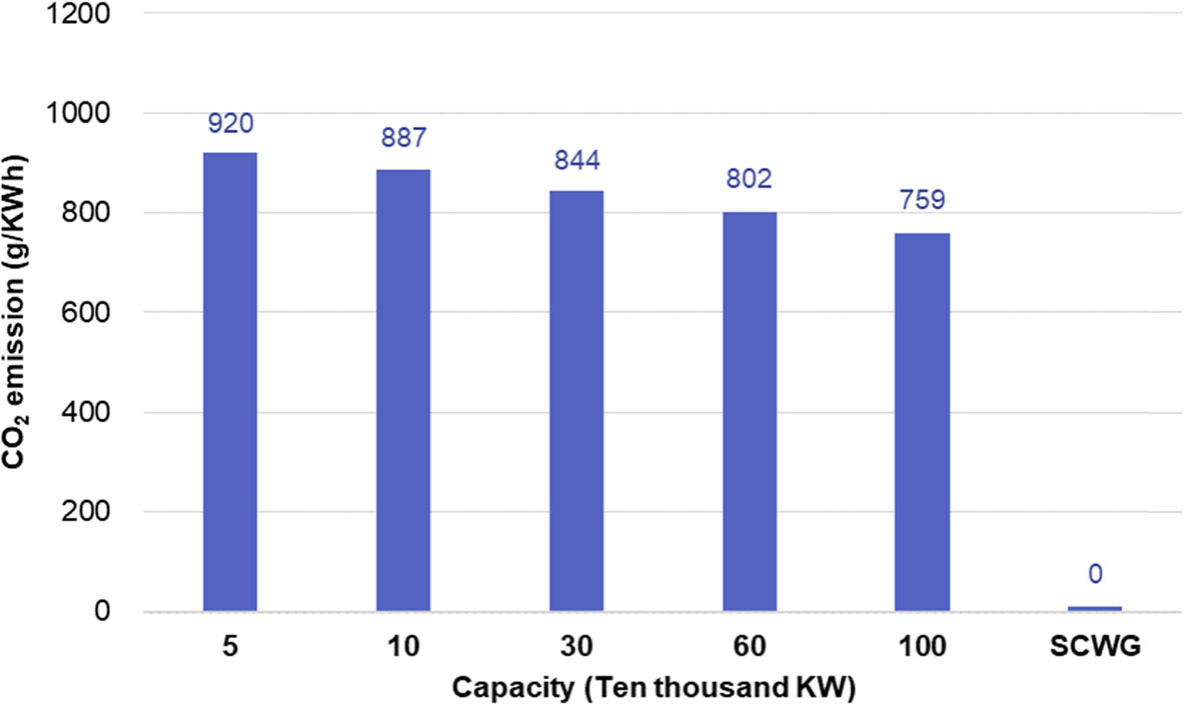
Comparison of SCWG and traditional technology on CO_2_ emission

Compared with coal-fired power generation, the SCWG-based technique shows overwhelming advantages in CO_2_ emission, as shown in Fig. 15. The high purity CO_2_ in the product can be easily collected and used as the resource for fuel production, achieving zero CO_2_ emission in coal utilization. Therefore, the SCWG-based technology can provide an effective way for China to achieve carbon mitigation goals.

#### Poly-generation for power generation and ammonia production

**Fig. 16:**
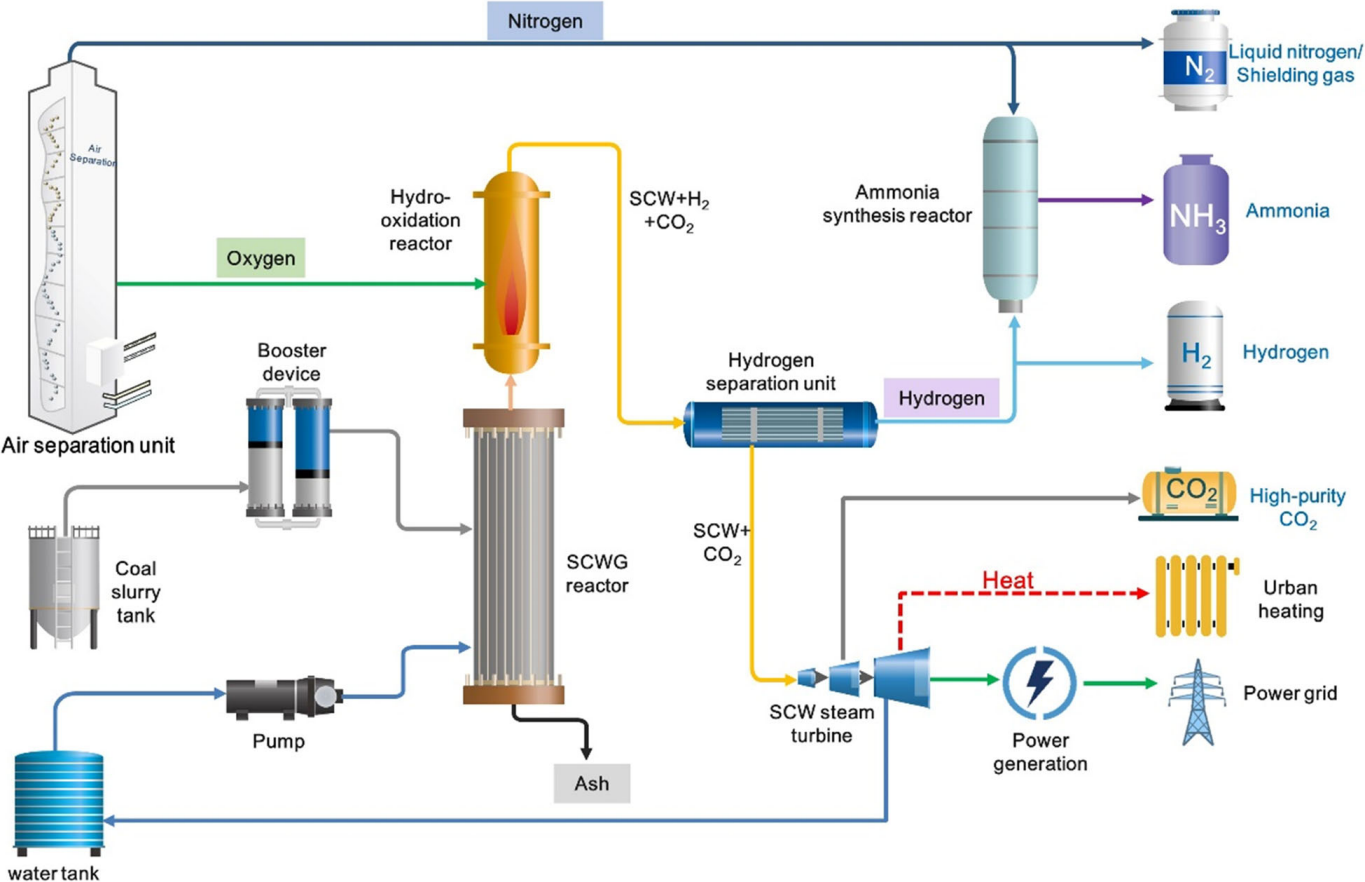
Schematic of poly-generation system for ammonia and hydrogen production

The SCWG-based technique can be designed to integrate with air separation processes to create a poly-generation system for ammonia production, hydrogen production, heating, and power generation. As shown in Fig. 16, the oxygen produced from the air separation unit can be used for reaction with hydrogen-rich gas products to provide heat for the system and the city. The high-pressure hydrogen separated from the SCWG unit can be directly reacted with the nitrogen (produced by the air separation unit) for ammonia synthesis, thus avoiding the compression costs in traditional coal chemical industry. The high-purity CO_2_ can be obtained through separating the gas products. As a result, the poly-generation system can efficiently realize the production of multiple gas products, including nitrogen, ammonia, hydrogen, and CO_2_. In addition, the mixed working medium produced from the system can be used for heat and power generation.

The poly-generation system enables the comprehensive utilization of resources and by-products, and many other attractive possibilities can be achieved. For example, the above processes can be achieved through treating other carbon-containing resources (e.g., biomass, crude oil, and wastes). Besides, it can be easily combined with traditional industries, such as for industrial and civil boiler replacement, for heat production, and so on. More importantly, the competitiveness in economic and environmental benefits enable the development and construction of a hydrogen-based energy utilization systems in a wide range of energy-related sectors, promoting the synergistic carbon reduction in the whole industrial chains.

## Solar fuel generation from water and CO_2_: large-scale solar energy utilization and carbon recycling

### The basic science of CO_2_RR powered by solar energy

**Fig. 17:**
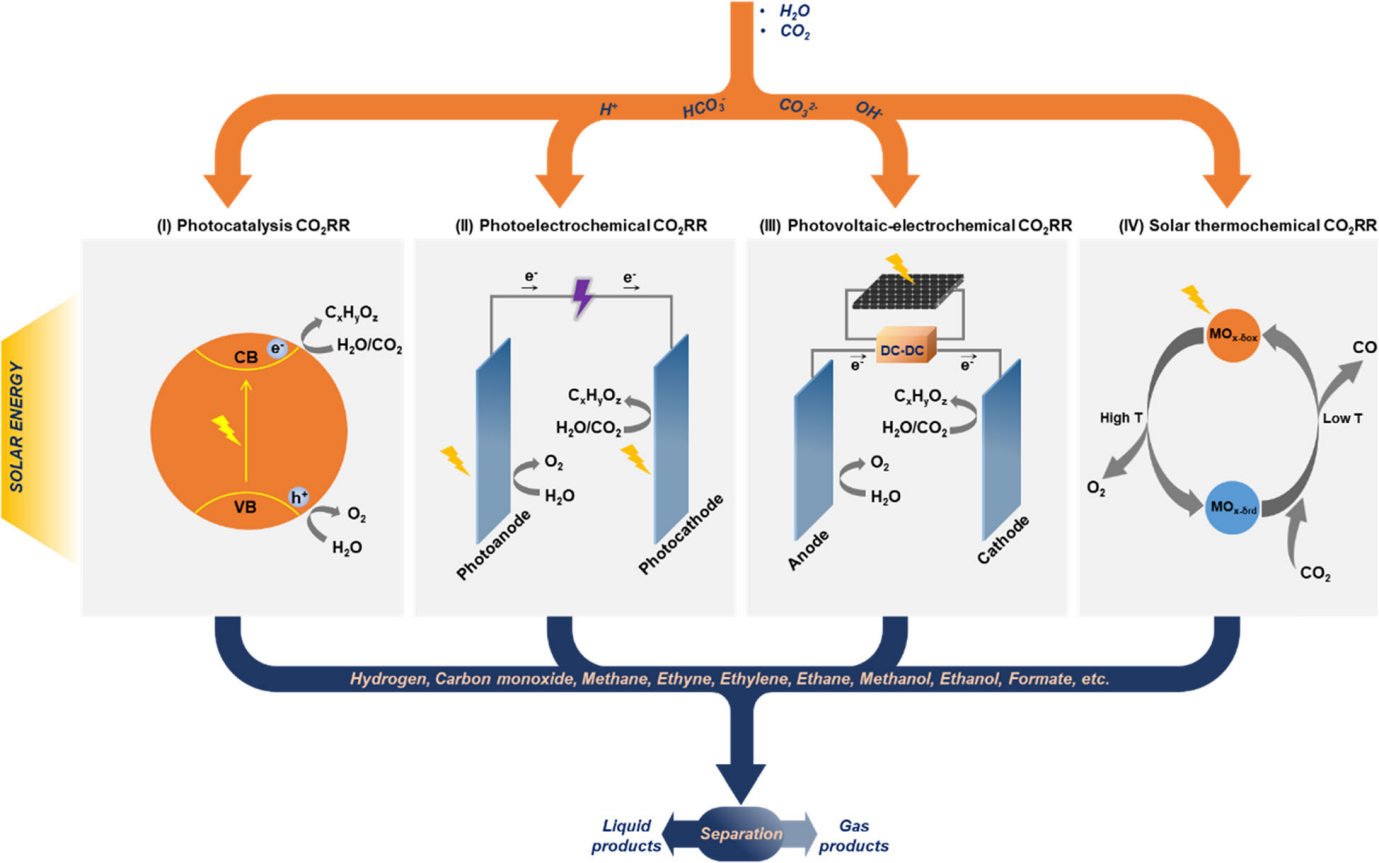
Schematic diagram of the current techniques for solar-driven CO_2_RR. With solar energy, H_2_O and CO_2_ can be reduced to various chemicals via photocatalysis, photoelectrochemical, photovoltaic-electrochemical, or solar thermochemical approaches

By using CO_2_RR techniques, CO_2_, and water (the waste in supercritical water gasification of coal) can be used to produce valuable carbon-based fuels or organic chemicals [110]. At present, solar-driven CO_2_RR techniques include photocatalysis, photoelectrochemical (PEC), photovoltaic-electrochemical (PV-EC), and solar thermochemical approaches, as shown in Fig. 17. Within all of the approaches, some small molecular compounds can be produced from a series of reduction reactions, where CO_2_RR exists. The identified products have been reported in the literature, including almost all C1, C2, and C3 products (for example CO, CH_4_, HCOOH, HCHO, CH_3_OH, CH_3_CH_2_OH, CH_3_COOH, C_2_H_4_, and so forth) as well as hydrogen. The thermodynamic equilibrium potentials, E^0^, of the chemicals are listed in Table 2.

**Table 2:** The thermodynamic equilibrium potentials E^0^, and products involved in CO_2_RR. E^0^ relative to the Standard Hydrogen Electrode (SHE) at pH = 0 is estimated from standard Gibbs free energy

Reactions	E^0^	Products
2H^+^_(aq)_ + 2e^−^ → H_2(g)_	0.00	Hydrogen
CO_2(g)_ + 2H^+^_(aq)_ + 2e^−^ → CO_(g)_ + H_2_O_(l)_	−0.12	Carbon monoxide
CO_2(g)_ + 2H^+^_(aq)_ + 2e^−^ → HCOOH_(l)_	−0.20	Formic acid
CO_2(g)_ + 4H^+^_(aq)_ + 4e^−^ → CH_2_O_(l)_ + H_2_O_(l)_	−0.07	Formaldehyde
CO_2(g)_ + 6H^+^_(aq)_ + 6e^−^ → CH_3_OH_(l)_ + H_2_O_(l)_	0.03	Methanol
CO_2(g)_ + 8H^+^_(aq)_ + 8e^−^ → CH_4(g)_ + 2H_2_O_(l)_	0.17	Methane
2CO_2(g)_ + 2H^+^_(aq)_ + 2e^−^ → H_2_C_2_O_4(aq)_	− 0.50	Oxalic acid
2CO_2(g)_ + 12H^+^_(aq)_ + 12e^−^ → CH_2_CH_2(g)_ + 4H_2_O_(l)_	0.06	Ethylene
2CO_2(g)_ + 12H^+^_(aq)_ + 12e^−^ → CH_3_CH_2_OH_(l)_ + 3H_2_O_(l)_	0.08	Ethanol
2CO_2(g)_ + 14H^+^_(aq)_ + 14e^−^ → CH_3_CH_3(l)_ + 4H_2_O_(l)_	0.14	Ethane

**Fig. 18:**
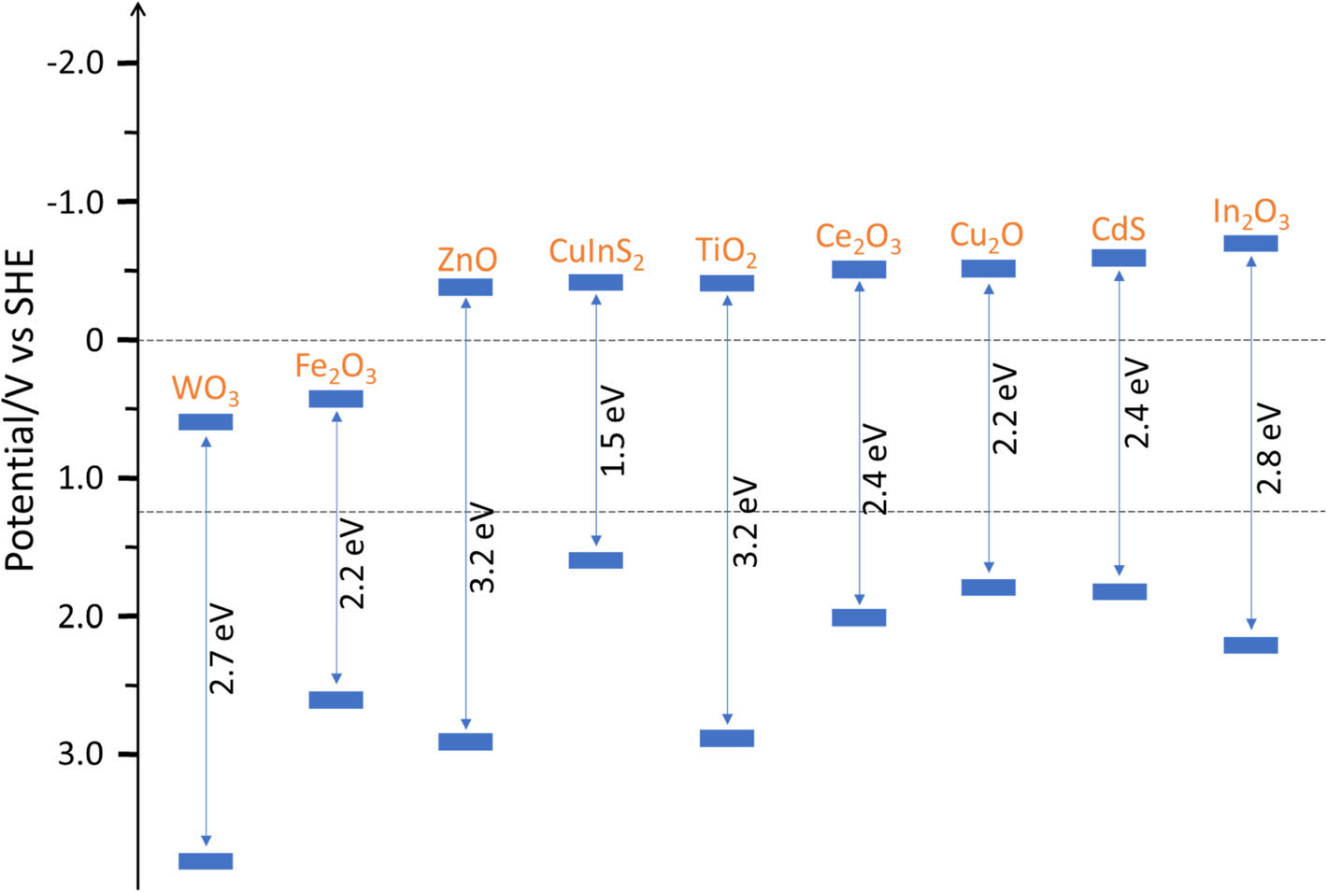
Band positions of typical inorganic photocatalyst materials relative to SHE

In a photocatalysis approach, CO_2_RR is powered by the solar-generated electron-hole pairs when the photocatalyst absorbs solar energy. Figure 18 presents the band positions of typical semiconductors which own appreciable photocatalysis activity. The photocatalyst can not only convert the light energy into photovoltage, but also lower the activation energy of the chemical reactions. As shown in Fig. 17, within photocatalysts, solar energy excites electrons from the valence band (VB) to the conduction band (CB). The photogenerated electrons and the holes left participate in the CO_2_RR and oxygen evolution reaction (OER), respectively, to generate abundant fuels and chemicals. Owing to the bandgap, the semiconductor can absorb solar energy in the appropriate spectral range. The semiconductors with good light adsorption effects have received widespread attention from all walks of life and have achieved rapid development. In recent years, Z-scheme, which is constructed by two materials with different bandgaps, can make up for the shortcoming of a single semiconductor. A representative work of this system has achieved 896.7 and 440.7 μmol/(g∙h) of formate and oxygen at a stoichiometric ratio [111]. Domen and coworkers constructed RuO_x_/Mo:BiVO_4_-charge mediator-Ru/La,Rh:SrTiO_3_ Z-scheme composite photocatalyst to achieve a high energy conversion efficiency of hydrogen and formate [112].

The typical PEC-CO_2_RR system has four parts: photoelectrode, membrane, electrolyte, and external circuit. Other than solar energy, PEC systems can also accept a supporting external bias [113]. Solar-driven electron-hole pairs are generated on photoelectrodes (photocathode or photoanode), and then produce the photocurrent continuously within the closed circuit. In this process, CO_2_RR would occur on the surfaces of the (photo)cathode, while oxygen evolution reaction (OER) occurs on the surfaces of the (photo)anode. Over the past few decades, solar energy utilization and fuel production system based on PEC has attracted researchers’ interest and many materials have been explored to manufacture PEC photoelectrodes. So far, the highest energy conversion efficiency using PEC for CO_2_RR comes from Xiang’s group. They use GaAs/InGaP/TiO_2_/Ni photoanode and series Pd/C photocathode to achieve 10% solar-to-fuel (STF) and > 94% Faradaic efficiency of formate [114].

PV-EC is a combination of photoelectric conversion and electrochemical CO_2_RR. As a light energy absorber, PV absorbs photons and generates electron carriers, which are transmitted to the electrolytic cell through external wires, providing all the electrical energy for the oxidation-reduction reaction of the positive and negative electrodes of the electrolytic cell. It is independent of the electrochemical cell and does not come into contact with the electrolyte, so it has excellent stability compared with the integrated photoelectrode in PEC systems. A high-efficient PV would promote PV-EC systems to achieve higher STF efficiency. Furthermore, the chemical reactions that occurred on PV-EC take place under normal temperature and pressure without additional energy input. So far, the highest energy conversion efficiency using PV + EC for CO_2_RR comes from Lin’s group. They use NiFe/NF photoanode and Ag photocathode to achieve ~ 23.4% STF and > 99% Faradaic efficiency of CO [115].

In general, the solar thermochemical system consists of two steps, which are separated in time and space [116]. In the first step, the catalyst releases oxygen at a relatively high temperature (~ 1400 °C), and thereby shows reduction ability. In the second step, part of oxygen is taken from CO_2_ by the catalyst, producing CO. Although in theory, the STF by photothermal catalysis could reach considerable efficiency [117], the cost of creating ultra-high reaction temperature through large-scale concentration of light still restricts the industrial use of this technology. Using reticulated ceria foams as oxygen carrier material and volumetric porous solar absorber, Haeussler et al. designed a novel monolithic solar reactor for two-step H_2_O and CO_2_ splitting and achieved the highest STF efficiency of 7.5% for producing H_2_ and CO [118].

### Industry development status in CO_2_ reduction

It is a promising way that couple the CO_2_ reduction system with the SCWG system, to achieve the goal of peaking carbon emission and carbon neutrality. There is a large amount of CO_2_ and H_2_O in the SCWG product, which can be directly injected into the CO_2_RR system as reactants after cooling, and thermal energy, which can be used in photothermal catalysis [119, 120]. This way can be a solution for the dual purpose of removing CO_2_ and producing hydrocarbon products, and then achieving the goal of fixing carbon. At present, the role of the global energy transition has attracted more and more attention from the international community. Renewable energy has become an important part of the future energy strategy, the core of energy transition, and the new platform for job creating and economic growth. To date, solar energy has become the fourth largest source of power generation after coal, natural gas, and hydropower. In 2022, the newly global installed capacity of photovoltaic is expected to reach 190 GW and by 2032, wind and solar power generation are expected to exceed coal-fired power generation. The proportion of renewable energy in global power generation will increase exponentially. The United States, Germany, Denmark, and other countries have proposed the proportion of renewable energy to be 80% to 100% of electricity consumption by 2050.

To this end, many projects for the use of renewable energy have been initiated. The RECODE project of the Italian Institute of Technology focused on CO_2_ recovery and utilization process within the cement industry. The tail-carbon dioxide flue gas (25% by volume) from the cement manufacturing process is used to produce value-added chemicals and materials. The Politecnico di Torino developed a PEC reactor that uses water and sunlight to convert CO_2_ to methanol in the Eco2CO2 project, achieving a conversion efficiency of more than 6% under sunlight above 400 nm wavelength and continuous operation more than 10,000 h. Based on this technology, the annual reduction of up to 50 tons of CO_2_ emissions per year has been put on the plan. The European Commission’s project CEOPS is also focused on sustainable methods of producing methanol from CO_2_RR. Two chemical pathways were proposed in this program, CO_2_ to CH_4_ and CH_4_ to CH_3_OH, with methane as the intermediate carbon carrier. To improve their efficiency in both pathways, CEOPS has studied advanced catalysts in three promising electrocatalytic processes: dielectric-barrier discharge plasma catalysis, photoexcited catalysis, and electrocatalysis. The European Institute of Catalysis has teamed up with GASKATEL to develop an integrated process, in which high-value C2 chemicals can be produced from CO_2_ using electrochemical technology in the OCEAN project. By matching the dynamic process on anode and cathode, 250 g of CO_2_ can be converted per hour at the current density of 1.5 kA/m^2^, which reduces the economic cost in the process of CO_2_RR. The SunCoChem project catalyzes the carbonylation of C-C bonds through an enhanced coupling of solar-powered CO_2_RR to CO and water oxidation to O_2_ with a novel multi-functional hybrid photocatalyst [114, 121]. The project will improve the catalytic performance of the materials which are potential and earth-rich. By coupling renewable solar energy with a carbon source (CO_2_), carbon neutrality between energy and high-value chemicals could be achieved, which meets European dependence on carbon materials in the chemical industry.

### Vision for the industrialization indicator and economic analysis

CO_2_RR makes renewable energy stored in the form of chemicals on a large scale. Techno-economic analysis (TEA) can be used to assess CO_2_ conversion processes, the feasibility of obtaining economical products, and further determine the indicators for evaluating performance. Market size is critical to guide product selectivity. In this respect, methane, methanol, ethanol, and ethylene are promising products, because each of them has a market demand more than 80 million tons per year. Methane is the main component of natural gas and a precursor for various chemicals. Methanol and ethanol are used as solvents, precursors, and direct fuels. Ethylene is an important precursor in the polymer industry, especially in the synthesis of polyethylene.

The cost of CO_2_RR products takes both capital cost and operating cost into account. Capital costs come from CO_2_ electrolyzer while operating costs come from power usage, CO_2_ feedstock, and product separation costs. In our coupled system, the overall cost is reduced by cutting the CO_2_ feedstock and part of the electric power from renewable energy. Sargent et al. estimated the cost of CO_2_ electrolyzers by analogy with water electrolyzers [122]. The cost of CO_2_ electrolyzers would be 5000–15,000 $/m^2^ in the absence of large-scale commercial operation, which is the same as the cost of proton exchange membrane (PEM) water electrolyzers. Furthermore, product separation accounts for a large proportion of the cost. For gas product separation, pressure swing adsorption (PSA) and membrane technologies are being used in some other industrial processes with a similar gas composition. The selected separation cost is 10 $/t, which is equivalent to the cost of industrial-based biogas separation technologies. In addition, liquid products separation can be achieved by distillation, extraction, precipitation, and pervaporation. Compared to the gas separation by using PSA, the capital cost of liquid separation is similar, but the operational cost is much higher. According to the Sherwood diagram, the separation cost of the liquid product is estimated to be 60 $/t, assuming the minimum input product concentration is 10% [123].

In industrial applications, the rate of chemical production (yield) is the primary criterion to ensure profitability. The current density, which reflects the reaction rate, directly affects the cost of capital. Using alkaline electrolytic cell with a cost of 920 $/m^2^, Jouny estimated that a current density of 250–300 mA cm^− 2^ is feasible. Besides, Faraday efficiency (FE), energy efficiency (EE), and stability are also used to characterize the performance of the process. FE reflects the selectivity of the current to a specific CO_2_RR product. The high FE reduces the separation requirements and the total current required for the target production rate. EE is the percentage of the energy stored in the desired product to the total energy required to synthesize them. The electricity used is proportional to EE and the product energy value. Improving overall energy efficiency can reduce energy inputs and costs. Finally, CO_2_ electrolyzer should achieve a long duration under production conditions. The industrial water electrolytic cell for reference has been running stably for more than 80 thousand hours. Long-term stability is essential to reduce maintenance, replacement costs and associated with cell downtime. Sargent et al. put forward that the target performance indicators of current density (> 300 mA·cm^− 2^), FE (80–90%), battery voltage (< 1.8 V), and stability (> 80,000 h) need to be achieved for CO_2_RR to be economically viable [122].

There are many scientific and engineering challenges for this technology to be truly used in the industrial market, with the development of society and the emergence of new modes of operation, the opportunity of the renewable energy market is likely to arise. In addition to the technical challenges, there are considerable economic barriers in the complex, mature, and highly interconnected petrochemical industry. Despite these challenges, the development and adoption of renewable energy technologies, such as solar and wind power, still provide a promising way for carbon neutrality.

## Perspective

The SCWG-based technology shows economic advantages in hydrogen production, heat supply, power generation, and potentially in ammonia production. At present, the technological feasibilities of SCWG have been widely proved through basically theoretical research, lab-scale experimental study, and even pilot scale testing. The mild operating parameters make it possible to use normal and cheap steels for equipment construction. There may still lie some hidden problems which can only be revealed through industrial-scale demonstration projects. Corresponding work will obviously lead to much more investments, and is the subject of our current work. Besides, a lot of new possibilities can be discovered, as the whole society is undergoing profound changes towards a green and sustainable future: the widespread renewable utilization is required, and using hydrogen as the energy carrier for end-use is promising. We aim at meeting these urgent demands from industrials through validating, optimizing, and advancing the SCWG system. We call for more participation and social support to push this promising technology towards industrial applications.

**Fig. 19:**
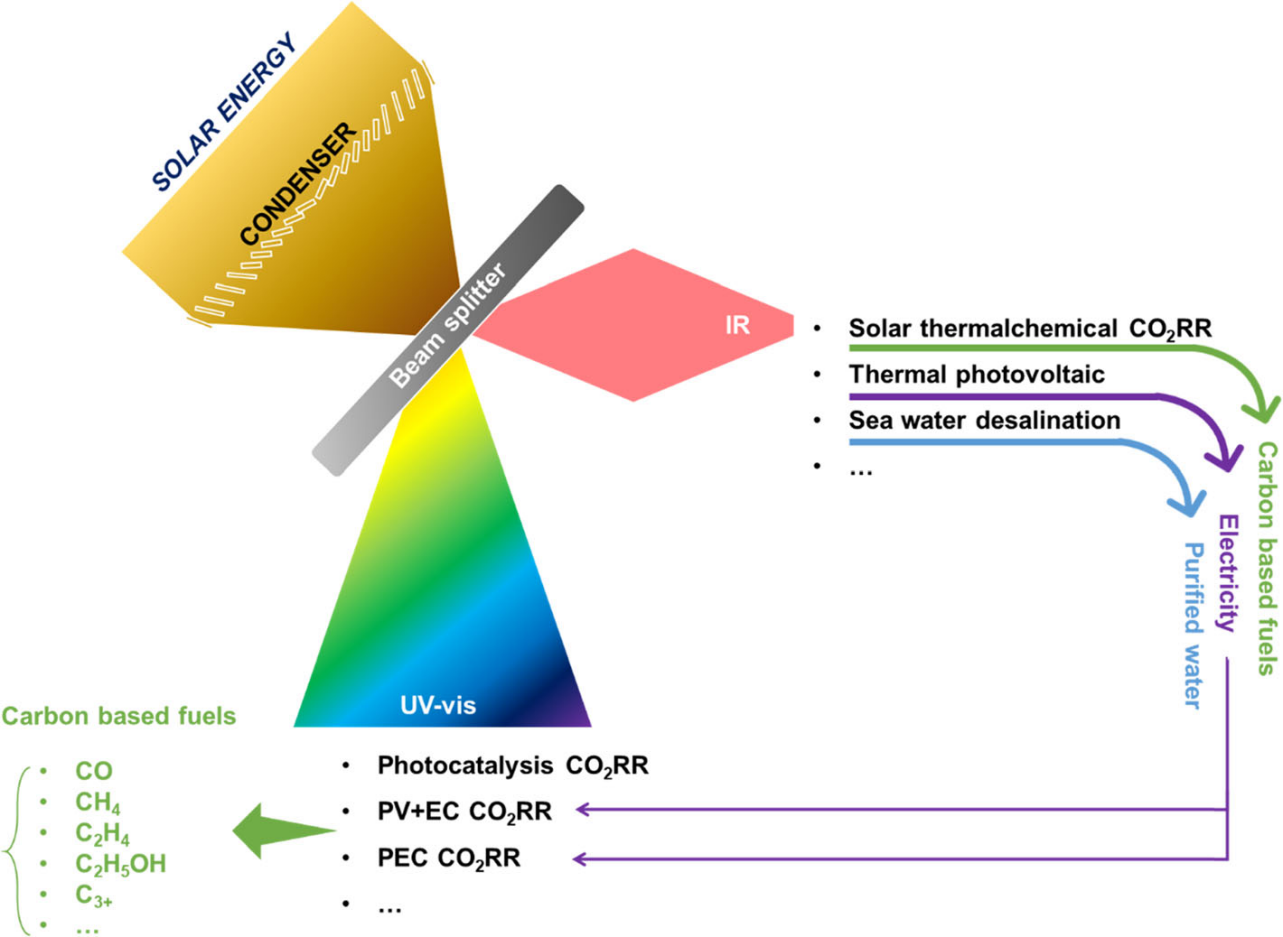
Scheme depicting a conceptually poly-generation technology in SKLMFPE

According to the technical route for preparing hydrocarbon fuels by solar energy with CO_2_RR technology, photocatalysis, PEC, and PV-EC systems can only utilize ultraviolet and visible (UV-vis) light. Although PEC and PV-EC systems own a higher STF efficiency than other solar fuel generation approaches, nearly all infrared (IR) light is wasted. Within the solar thermochemical system, all of the absorbed light is just for heating. Therefore, the combination of two or more solar fuel generation approaches should be a good choice for gradient utilization of the full spectrum. As shown in Fig. 19, solar light can be divided into UV-vis light and IR light by a beam splitter (or other wavelength dividers). UV-vis light could be used to drive photocatalysis, PEC, or PV-EC systems. While IR light can be used for the systems such as synthesizing carbon-based fuels via solar thermochemical CO_2_RR, generating electricity via seawater desalination, producing CO_2_ pyrolysis, and so on. In this case, besides carbon-based fuels, a greater variety of products (purified water, electricity, and so on) can be obtained simultaneously.

**Fig. 20:**
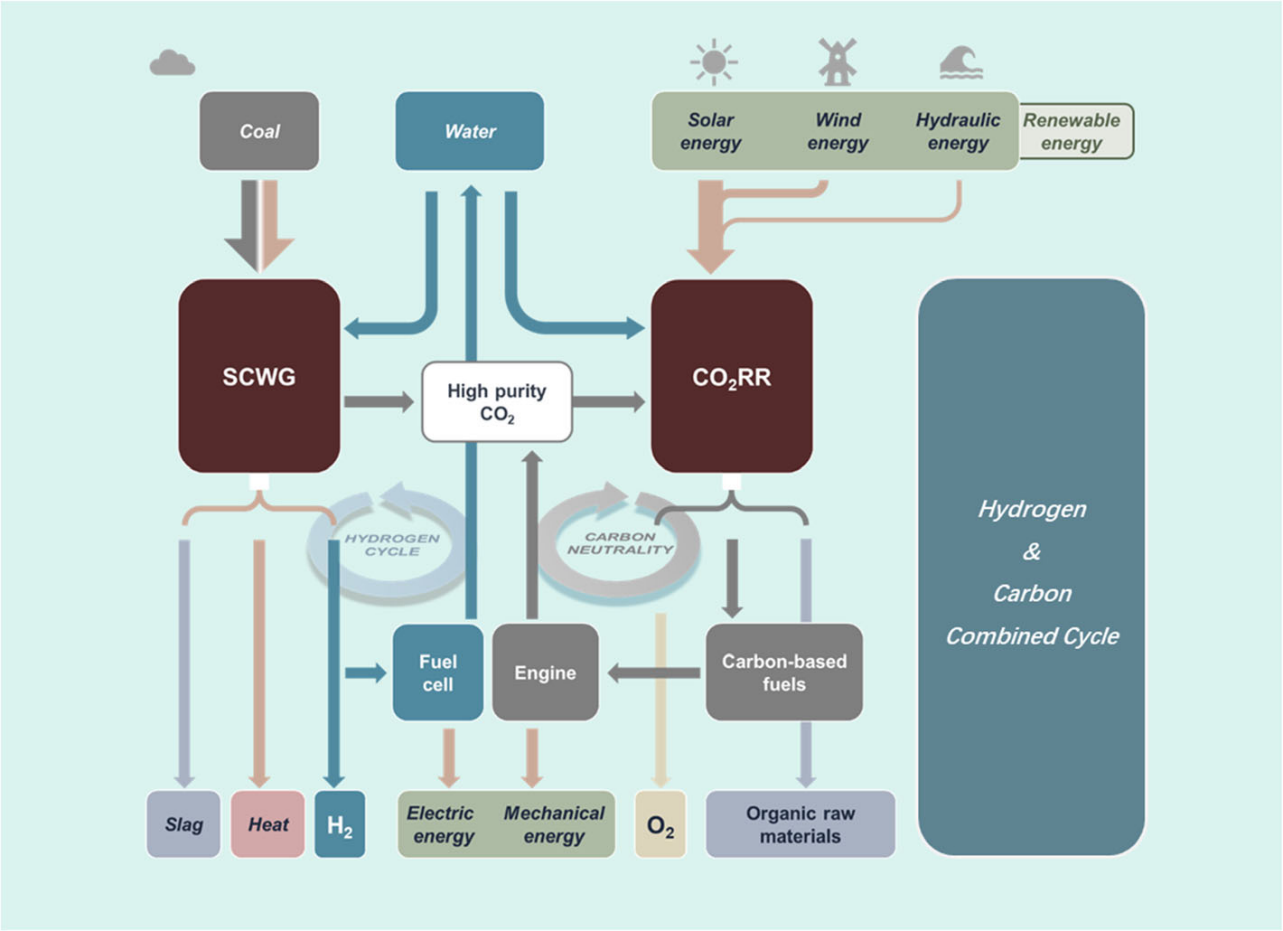
Scheme depicting identified Hydrogen & Carbon Combined Cycle (HCCC) for producing electric energy, mechanical energy, heating, and chemicals (H_2_, O_2_, and organic raw materials)

Through combing the SCWG-based coal conversion process with the renewables-powered CO_2_RR process, we propose an identified Hydrogen & Carbon Combined Cycle (HCCC) system, as shown in Fig. 20. Being powered by primary energies, such as coal, solar energy, wind energy, and/or hydraulic energy, HCCC produces electric energy, mechanical energy, heat, and chemicals in an environmentally friendly way. In this system, coal brings both energy and carbon into the SCWG reactor, which produces slag, heat, H_2_, as well as high-concentration CO_2_ for the following CO_2_RR reactor. Next, renewable energies (solar energy, wind energy, and hydraulic energy) can be transformed to clean and pollution-free hydrocarbon fuels and valuable chemical products which are convenient to store and transportation. Also, O_2_ can be a by-product. After being used for generating electric energy and mechanical energy, the generated H_2_ and carbon-based fuels are further converted to water and CO_2_, which can be used as raw materials for SCWG and CO_2_RR. In addition, H_2_ is an important raw material for synthesizing chemical compounds and in metallurgical reduction reactions, other than an energy carrier.

The whole process within the HCCC system does not create any carbon emissions. Compared to the current industrial system, HCCC eliminates the urgent issues in (i) reducing CO_2_ emissions in coal-fired power plants and industrial coal consumption; and (ii) solving instabilities issues to the electrical grid caused by the intermittence of renewable power. As the commitment announced by the Chinese government in “Climate Ambition Summit 2020”, which aims to increase the share of non-fossil fuels in primary energy consumption to around 25% in 2030, HCCC can not only reduce fossil fuel consumption and increase the proportion of renewable energy applications, but also provide a promising way to utilize renewable energy sources including but not limited to solar energy, wind energy and hydraulic resources to valuable chemical products. The quantitative match between SCWG and CO_2_RR still requires much work to do, which is one of our future research focuses.

## Conclusion

In the context of carbon mitigation, the huge amount of CO_2_ emission from a wide energy-related industry, mainly from fossil consumption, should be directly mitigated. SCWG-based technology can directly mitigate the CO_2_ emission through subverting the traditional coal utilization mode: it orderly converts the chemical energy of coal and low-grade heat into hydrogen and CO_2_ in a naturally captured state; the renewables-powered CO_2_ reduction techniques further convert the high-purity CO_2_ into carbon-based fuels, leading to zero carbon emission.

Besides, the SCWG process provides a promising way in meeting the wide demands from industries and in pushing the energy transition towards a green and sustainable future: 1) SCWG achieves the poly-generation of steam, heat, hydrogen, electricity, CO_2_ and minerals, which can be easily combined with and upgrade the traditional industries; 2) the CO_2_RR achieves the effective conversion of intermittent renewable energy into stable chemical energy of carbon-based fuels, releasing the bottleneck of renewable development.

The SCWG-based coal utilization system achieves the efficient energy conversion and full utilization of resources without CO_2_ and pollutant emission, exemplifying the physiologic of ordered energy conversion: the energy conversion and resource utilization are combined in a desired way, in which the maximum energy efficiency can be achieved, and the substances are fully used as resources. The wealth of knowledge disseminated in this paper is not restricted to currently discussed technologies, but could inspire the whole energy-related industries to reconsider the energy utilization system towards an efficient and sustainable future.

## Data Availability

All data generated or analyzed during this study are included in this published article.

## Abbreviations

CB: Conduction band
CCS: Carbon capture and storage
CO_2_: Carbon dioxide
COP: Conference of the Parties
CO_2_R: CO_2_ reduction
CO_2_RR: CO_2_ reduction reaction
EE: Energy efficiency
FE: Faraday efficiency
H_2_: Hydrogen
HCCC: Hydrogen & Carbon Combined Cycle
HER: Hydrogen evolution reaction
IEA: International Energy Agency
IR: Infrared
NO_x_: Nitrogen oxide
OER: Oxygen evolution reaction
PAH: Polycyclic aromatic hydrocarbons
PEC: Photoelectrochemical
PEM: Proton exchange membrane
PM: Particulate matter
PSA: Pressure swing adsorption
PV-EC: Photovoltaic-electrochemical
SCW: Supercritical water
SCWG: Supercritical water gasification
SKLMFPE: State Key Laboratory of Multiphase Flow in Power Engineering
SO_x_: Sulfur oxide
STF: Solar-to-fuel
TEA: Techno-economic analysis
UV-vis: Ultraviolet and visible
VB: Valence band
